# The Dual Function of KDM5C in Both Gene Transcriptional Activation and Repression Promotes Breast Cancer Cell Growth and Tumorigenesis

**DOI:** 10.1002/advs.202004635

**Published:** 2021-02-18

**Authors:** Hai‐feng Shen, Wen‐juan Zhang, Ying Huang, Yao‐hui He, Guo‐sheng Hu, Lei Wang, Bing‐ling Peng, Jia Yi, Ting‐ting Li, Rui Rong, Xiao‐yan Chen, Jun‐yi Liu, Wen‐juan Li, Kenny Ohgi, Shao‐Wei Li, Michael G. Rosenfeld, Wen Liu

**Affiliations:** ^1^ State Key Laboratory of Cellular Stress Biology Fujian Provincial Key Laboratory of Innovative Drug Target Research School of Pharmaceutical Sciences Xiamen University Xiang'an South Road Xiamen Fujian 361102 China; ^2^ State Key Laboratory of Molecular Vaccinology and Molecular Diagnostics Xiamen University Xiang'an South Road Xiamen Fujian 361102 China; ^3^ School of Life Sciences Xiamen University Xiang'an South Road Xiamen Fujian 361102 China; ^4^ Howard Hughes Medical Institute Department of Medicine University of California 9500 Gilman Drive La Jolla San Diego CA 92093 USA

**Keywords:** breast cancer, estrogen and estrogen receptor, Jumonji C domain‐containing protein, type I interferon

## Abstract

Emerging evidence suggested that epigenetic regulators can exhibit both activator and repressor activities in gene transcriptional regulation and disease development, such as cancer. However, how these dual activities are regulated and coordinated in specific cellular contexts remains elusive. Here, it is reported that KDM5C, a repressive histone demethylase, unexpectedly activates estrogen receptor alpha (ER*α*)‐target genes, and meanwhile suppresses type I interferons (IFNs) and IFN‐stimulated genes (ISGs) to promote ER*α*‐positive breast cancer cell growth and tumorigenesis. KDM5C‐interacting protein, ZMYND8, is found to be involved in both processes. Mechanistically, KDM5C binds to active enhancers and recruits the P‐TEFb complex to activate ER*α*‐target genes, while inhibits TBK1 phosphorylation in the cytosol to repress type I IFNs and ISGs. Pharmacological inhibition of both ER*α* and KDM5C is effective in inhibiting cell growth and tumorigenesis. Taken together, it is revealed that the dual activator and repressor nature of an epigenetic regulator together contributes to cancer development.

## Graphical abstract

A histone demethylase named KDM5C activates estrogen/estrogen receptor alpha‐target genes to promote cancer cell growth, while suppresses type I interferons and interferon‐stimulated genes to escape from immune surveillance. The dual activator and repressor nature of KDM5C together contributes to cancer development.

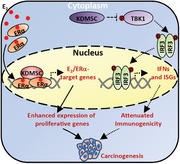

## 1 Introduction

In general, breast cancers are classified into five subtypes based on the expression of estrogen receptor (ER), progesterone receptor (PR), and human epidermal growth factor receptor 2 (HER2), which are luminal A, luminal B, HER2‐enriched, basal‐like, and normal‐like breast cancer subtypes.^[^ ^1^ ^]^ Both luminal A and B subtypes are ER expression‐positive, which account for around two thirds of all breast cancer subtypes. ERs, both estrogen receptor alpha (ER*α*) and ER*β*, belong to a family of ligand‐activated transcription factors, which are activated by steroid hormone estrogens (17‐*β*‐estradiol, estradiol, E_2_). Specifically, estrogen binds to ER and induces the translocation of ER from cytosol to nucleus, where ER binds to chromatin DNA in *trans* or in *cis* through a consensus estrogen response element (ERE), and further recruits a large number of co‐activators to its target genes’ promoter and/or enhancer regions to activate genes with implications in cell cycle regulation, cell metabolism, and immune response, among others.^[^ ^2^ ^]^ The prolonged exposure to high levels of estrogen will lead to the constitutive activation of estrogen/ER‐regulated gene program, which has been shown to be one of the major causes of ER‐positive breast cancers.

Cyclic GMP‐AMP synthase (cGAS) is a pattern recognition receptor that can sense cytosolic DNA including those derived from mitochondria, nuclear DNA leakage, micro‐nuclei, and other sources such as pathogenic viruses.^[^ ^3^ ^]^ Upon DNA binding, cGAS undergoes conformational changes that allow cGAS to catalyze ATP and GTP into 2′3′‐cyclic GMP‐AMP (cGAMP), which functions as a second messenger and binds to adaptor protein STING.^[^ ^4^ ^]^ The binding of cGAMP induces a conformational change in STING, exposing the carboxyl (C) ‐terminus for TBK1 binding and activation.^[^ ^5^, ^6^ ^]^ TBK1 further phosphorylates IRF3, which translocates into the nucleus to induce the expression of type I interferons (IFNs).^[^ ^5^, ^7^ ^]^ Type I IFNs bind to the type I IFN receptor, which activates a signaling cascade, leading to the expression of a large number of IFN‐stimulated genes (ISGs).^[^ ^7^ ^]^ Type I IFNs and ISGs have been reported to facilitate cancer immune surveillance, antitumor immunity, and antitumor efficacy of immunotherapy.^[^ ^8^ ^]^ Tumor cells often develop mechanisms to suppress the cGAS‐STING‐TBK1 signaling pathway and therefore the production of type I IFNs and ISGs, escaping from immune surveillance and antitumor treatment such as immunotherapy.^[^ ^8^ ^]^

KDM5C, also named as JARID1C or SMCX, belongs to the KDM5 subfamily of JmjC (Jumonji C) domain‐containing histone demethylases, which catalyzes the removal of the methyl groups from di‐ and tri‐methylated lysine 4 on histone H3 (H3K4me2/3) in an Fe (II)‐ and *α*‐ketoglutarate‐dependent manner.^[^ ^9^, ^10^, ^11^ ^]^ Other KDM5 family members, including KDM5A (JARID1A/RBP2), KDM5B (JARID1B/PLU‐1), and KDM5D (JARID1D/SMCY), also demethylate H3K4me2/3.^[^ ^10^, ^11^ ^]^ KDM5C contains a JmjN domain, a BRIGHT domain, an ARID domain (AT‐rich interaction domain), a JmjC domain, a C5HC2 zinc finger, and two PHD (plant homeodomain) fingers. KDM5C binds to tri‐methylated lysine 9 on histone H3 (H3K9me3) via its N‐terminal PHD finger, which is required for it to efficiently demethylate H3K4me2/3 and repress gene transcription.^[^ ^10^ ^]^ KDM5C has been shown to play an essential role in neurogenesis, DNA damage response, and cancer development, among others. Nonsense, missense, and frameshift mutations were often found in KDM5C gene in families with X‐linked mental retardation (XLMR)^[^ ^12^ ^]^ and autism spectrum disorder.^[^ ^13^ ^]^ Notably, most of these mutations impaired KDM5C's demethylase activity toward H3K4me2/3 and therefore KDM5C‐mediated transcriptional repression of neuronal genes, suggesting that KDM5C might contribute to XLMR.^[^ ^10^, ^11^, ^14^ ^]^ Indeed, disruption of the mouse KDM5C gene recapitulates adaptive and cognitive abnormalities observed in XLMR, suggesting that KDM5C mutations are causal to XLMR.^[^ ^15^ ^]^ However, there are also mutations, such as R1115H mutation, reported to have no impact on KDM5C demethylase activity, but they failed to repress target gene expression as efficiently as wild‐type (WT) KDM5C, suggesting that KDM5C can also regulate gene transcription and contribute to XLMR in an enzymatic‐independent manner.^[^ ^16^ ^]^ Besides its well‐recognized role in neurogenesis, KDM5C was found to be tightly associated with various types of cancers. In clear cell renal cell carcinoma, inactivating mutations were found in KDM5C, which led to the aberrant activation of heterochromatin non‐coding RNAs that subsequently triggered genomic instability.^[^ ^17^ ^]^ In breast cancers, KDM5C was found to be significantly upregulated compared to normal breast tissues and positively correlated with metastasis. Silencing of KDM5C in breast cancer cells inhibited cell migration and invasion, which was due to the release of repression on its target gene BRMS1, a metastasis suppressor.^[^ ^18^ ^]^ Recently, upregulated expression of KDM5C in breast cancer cells was reported to suppress the expression of STING and thus type I IFNs and ISGs, through which cells escape from immuno‐surveillance. Accordingly, KDM5C expression is inversely associated with patient survival in multiple cancer types including breast cancer.^[^ ^19^ ^]^ In contrast, Shen et al. reported that KDM5C might function as a tumor suppressor by preventing hyperactivation of enhancers associated with oncogenes, such as S100A family genes. KDM5C loss led to increased cell migration and invasion in ZR‐75‐30 breast cancer cells.^[^ ^20^ ^]^ Another member in the KDM5 family proteins, KDM5B, was found to be critical for the development of the normal mammary gland and the growth of ER‐positive breast cancer cells.^[^ ^21^ ^]^ Accordingly, KDM5B is frequently amplified and overexpressed in ER‐positive breast tumors, which is associated with poor outcome in patients.^[^ ^22^ ^]^ The oncogenic role of KDM5 family proteins, particularly KDM5C, was also observed in gastric cancer,^[^ ^23^ ^]^ prostate cancer,^[^ ^24^ ^]^ and hepatocellular carcinoma,^[^ ^25^ ^]^ which is often linked to its demethylase activity targeting H3K4me3/2 and thus its co‐repressor function in gene transcriptional control. However, emerging evidence suggested that they can also function as transcriptional co‐activators. For instance, KDM5 family proteins are required for the activation of a large set of pro‐proliferative cell cycle genes and thus promote differentiation of white and brown pre‐adipocytes into mature adipocytes.^[^ ^26^ ^]^ KDM5A was found to form a complex with CLOCK‐BMAL1 to enhance Per2 and circadian gene transcription and regulate circadian rhythms in a demethylase‐independent manner. Mechanistically, KDM5A inhibits histone deacetylase 1 activity, leading to increased histone acetylation on Per2 promoter.^[^ ^27^ ^]^ The non‐redundant role in circadian oscillator function of KDM5A was further confirmed in Drosophila.^[^ ^27^ ^]^ Lid, the only KDM5 homolog in Drosophila, was also reported to function as a transcriptional co‐activator for dMyc‐induced gene expression, which was thought to due to its inhibitory effect on the deacetylase activity of Rpd3.^[^ ^28^, ^29^ ^]^ More recently, Lid was shown to directly regulate the expression of genes involved in mitochondrial structure and function, which is independent of KDM5's demethylase activity.^[^ ^30^ ^]^ However, how KDM5C's co‐repressor activity, namely its H3K4me2/3 demethylase activity, is masked and converted to a transcriptional co‐activator under certain circumstances remains largely unknown. Furthermore, whether and how the dual functions of KDM5C, both co‐activator and co‐repressor, are coordinated in cells remain uncharacterized.

The bromodomain protein ZMYND8, also called PRKCBP1, Spikar or RACK7, was first identified as an activated protein‐kinase‐C in human.^[^ ^31^ ^]^ It was later isolated as a debrin‐binding protein by a yeast two‐hybrid screening of a rat brain library.^[^ ^32^ ^]^ ZMYND8 contains a PHD finger, a BRD domain (bromodomain), a PWWP (Pro‐Trp‐Trp‐Pro) domain, and a MYND (Myeloid, Nervy and DEAF‐1) domain.^[^ ^33^ ^]^ The triple reader module (PHD‐BRD‐PWWP) at the amino (N)‐terminal of ZMYND8 is capable of interacting with DNA and simultaneously recognizing multiple histone post‐translational modifications.^[^ ^34^, ^35^ ^]^ ZMYND8 was found to be involved in the regulation of a variety of biological processes, such as neural differentiation, DNA damage response, and cancer development. Similar as KDM5C, ZMYND8 can act as both a co‐activator and a co‐repressor, and its function remains controversial. In regards to neuronal differentiation, ZMYND8 was found to interact with RCOR2 (REST Corepressor 2) to repress neuronal gene expression and neural differentiation in Xenopus,^[^ ^36^ ^]^ while it activated all‐trans retinoid acid (ATRA)‐induced neuronal genes in neuronal precursor cells.^[^ ^37^ ^]^ In prostate cancer, ZMYND8, together with KDM5D, acts as a transcriptional co‐repressor to repress the expression of metastasis‐linked genes, leading to decreased cellular invasiveness.^[^ ^35^ ^]^ However, an independent study demonstrated that ZMYND8 is oncogenic in prostate cancer such that it promotes angiogenesis in prostate cancer xenografts in zebrafish and tube formation in human HUVEC cultures largely through inducing VEGFA expression. In consistent with its oncogenic nature, ZMYND8 expression is upregulated in both prostate cancer xenografts in zebrafish and prostate cancer samples from patients.^[^ ^38^ ^]^ In breast cancer, ZMYND8, together with KDM5C, functions as a tumor suppressor by preventing hyperactivation of enhancers associated with oncogenes, such as S100A family genes, and suppresses anchorage‐independent growth, migration, and invasion abilities in vitro, as well as enhances tumor growth in a mouse xenograft model.^[^ ^20^ ^]^ In contrast, Chen et al. showed that ZMYND8 interacts with HIF‐1*α* and HIF‐2*α* and enhances elongation of HIF‐induced oncogenic genes in breast cancer cells. Genetic deletion of ZMYND8 decreases breast cancer cell colony formation, migration, and invasion in vitro, and inhibits breast tumor growth and metastasis to the lungs in mice.^[^ ^39^ ^]^ Accordingly, ZMYND8 is upregulated in human breast tumors and correlated with poor survival of patients with breast cancer.^[^ ^39^, ^40^ ^]^ More recently, it was reported that loss of ZMYND8 increases micronucleus formation and activates the cGAS‐STING signaling cascade to induce IFN*β* and ISGs in breast cancer cells and tumors.^[^ ^41^ ^]^ It is clear that ZMYND8 is often associated with KDM5 proteins, and they both can function as either a co‐activator or a co‐repressor, but it is unclear how the dual functions of ZMYND8 and KDM5 proteins are coordinated in the cell.

In the current study, through systematic screening of the histone demethylase family proteins, we identified KDM5C as one of the candidates which co‐activate estrogen/ER*α*‐driven gene transcription and promote the growth of ER*α*‐positive breast cancer cells. In the presence of estrogen, KDM5C was recruited to ER*α*‐bound active enhancers on chromatin, where its demethylase activity was masked by ER*α*, turning into a co‐activator by recruiting the P‐TEFb complex to activate estrogen/ER*α*‐target genes and promote cell growth. Meanwhile, KDM5C represses type I IFNs and ISGs by directly interfering TBK1 phosphorylation in an enzymatic‐dependent manner, protecting cells from immune surveillance. KDM5C‐interacting protein ZMYND8 appeared to be involved in both KDM5C‐activated and ‐repressed gene sets. Pharmacological inhibition of both ER*α* signaling and KDM5C was shown to be effective in inhibiting ER*α*‐positive breast cancer cell growth and tumorigenesis.

## 2 Results

### 2.1 KDM5C is Required for ER*α*‐Positive Breast Cancer Cell Proliferation and Tumorigenesis

**Figure 1:**
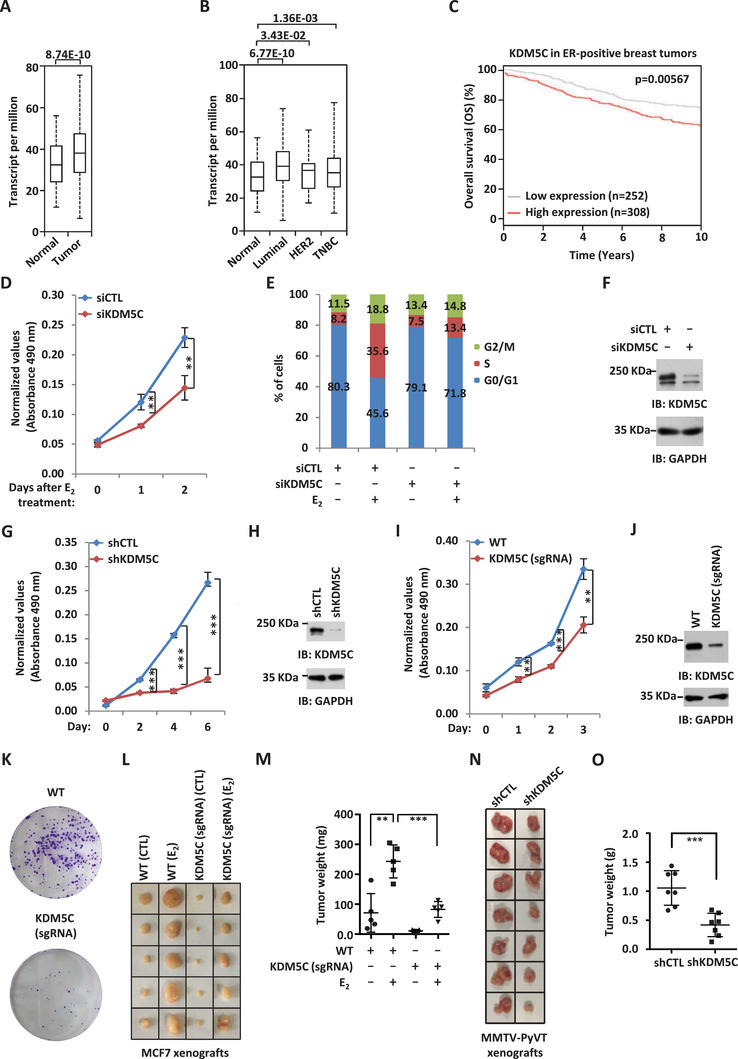
KDM5C is required for ER*α*‐positive breast cancer cell growth and tumorigenesis. A) The expression of KDM5C in a cohort of normal (*n =* 114) and clinical breast cancer (*n =* 1097) samples from TCGA (The Cancer Genome Atlas). B) The expression of KDM5C in different subtypes of clinical breast cancer samples from TCGA. (Normal, *n =* 114; Luminal, *n =* 566; HER2, *n =* 37; TNBC (triple‐negative breast cancer), *n =* 116). C) Kaplan–Meier survival analyses for OS (overall survival) of ER‐positive breast cancer patients using KDM5C as input (*n =* 560). D) MCF7 cells were transfected with control siRNA (siCTL) or siRNA specific against KDM5C (siKDM5C) in stripping medium for three days, and then treated with or without estrogen (E_2_, 10^−7^ m) for different duration as indicated followed by cell proliferation assay (± SD, ^**^ *p* < 0.01). E) MCF7 cells were transfected with siCTL or siKDM5C in stripping medium for three days, and then treated with or without estrogen (E_2_, 10^−7^ m) for 24 h followed by FACS analysis. F) MCF7 cells transfected with siCTL or siKDM5C were subjected to immunoblotting (IB) using antibodies as indicated. G) MCF7 cells were infected with control shRNA (shCTL) or shRNA specific against KDM5C (shKDM5C) lenti‐virus for duration as indicated followed by cell proliferation assay (± SD, ^***^ *p* < 0.001). H) MCF7 cells infected with shCTL or shKDM5C were subjected to immunoblotting (IB) using antibodies as indicated. I,K) Wild type (WT) and KDM5C knockdown (KDM5C (sgRNA)) MCF7 cells generated by CRISPR/Cas9 were subjected to cell proliferation assay (I) and colony formation assay (K) (± SD, ^**^ *p* < 0.01, ^***^ *p* < 0.001). J) WT and KDM5C (sgRNA) MCF7 cells were subjected to immunoblotting using antibodies as indicated. L) Xenograft experiments were performed by injecting WT and KDM5C (sgRNA) MCF7 cells subcutaneously into female BALB/C nude mice (5 mice per group) and then treated with or without estrogen (E_2_). Tumors were then excised, photographed, and weighted four weeks after subcutaneous injection. M) Weight of tumors as shown in (L) (± SD, ^**^ *p* < 0.01, ^***^ *p* < 0.001). N) Xenograft experiments were performed by injecting shRNA or shKDM5C lenti‐virus‐infected MMTV‐PyVT cells into female FVB mice (7 mice per group). Tumors were then excised, photographed, and weighted 23 days after subcutaneous injection. O) Weight of tumors as shown in (N) (± SD, ^***^ *p* < 0.001).

Histone demethylases, both LSD (LSD1 and LSD2) and JmjC domain‐containing protein families, have been well documented to be involved in cancer development. To identify histone demethylases which are required for the growth of ER*α*‐positive breast cancer cells, we transfected MCF7 cells, an ER*α*‐positive breast cancer cell line, with control siRNA or siRNA specifically against each individual member of the histone demethylase protein family except those that are specifically expressed on Y chromosome, then treated cells with or without estrogen followed by cell viability measurement. It was found that several demethylases were required for estrogen‐induced MCF7 cell growth, such as those in the KDM1, KDM4, KDM6, and KDM7 subfamilies, which was consistent with previous reports (Figure S1A, Supporting Information).^[^ ^42^ ^]^ The specificity and knockdown (KD) efficiency of the siRNAs used were extensively validated in our laboratory (data not shown). Out of all the demethylases that were found to be required for estrogen‐induced ER*α*‐positive breast cancer cell growth, KDM5C was particularly interesting partially due to its clinical relevance. Specifically, the expression of KDM5C was found to be significantly higher in clinical breast tumor samples than normal breast tissues (**Figure** **1**A). Furthermore, KDM5C appeared to have the highest expression in luminal (ER‐positive) subtypes (Figure 1B). High expression of KDM5C was correlated with poor prognosis in ER‐positive, but not ER‐negative, breast cancer patients (Figure 1C; Figure S1B, Supporting Information).

KDM5C's effects on MCF7 cell growth was reproducible (Figure 1D). In addition, KDM5C was found to promote cell growth in another two ER*α*‐positive breast cancer cell line, T47D and BT474 (Figure S1C,D, Supporting Information). Fluorescence‐activated cell sorting (FACS) analysis revealed that MCF7 cells were arrested at G1 phase when KDM5C was knocked down (Figure 1E). The KD efficiency of siRNA against KDM5C was examined by immunoblotting analysis (Figure 1F). KDM5C's effects on MCF7 cell growth and cell cycle progression were confirmed by an independent siRNA targeting KDM5C (Figure S1E–G, Supporting Information). Similar results were obtained with KDM5C KD by using two independent shRNAs (short hairpin RNA) or two independent sgRNAs (small guide RNA) (Figure 1G–J; Figure S1H–K, Supporting Information). KDM5C's effects on MCF7 cell growth were further demonstrated by colony formation assay (Figure 1K). To test whether KDM5C affects tumor growth in vivo, we injected nude mice subcutaneously with control MCF7 cells or MCF7 cells with KDM5C KD, and then treated with or without estrogen. Exogenous administration of estrogen was to promote the growth of tumor in mice. Tumor volume was dramatically induced after mice were estrogen‐treated for four weeks, which was significantly attenuated when KDM5C was knocked down (Figure 1L–M). We also injected mice with control MMTV‐PyVT cells or MMTV‐PyVT cells with KDM5C KD, and tumors were collected three weeks later. It was found that KDM5C KD led to a significant reduction of tumor volume, strengthening the oncogenic role of KDM5C in breast tumorigenesis (Figure 1N,O). Taken together, our data suggested that KDM5C is required for estrogen/ER*α*‐induced proliferation in cultured cells and tumorigenesis in xenograft mouse models, and the oncogenic role of KDM5C might be clinically relevant, with that it is highly expressed in breast tumor samples and associated with poor prognosis.

### 2.2 KDM5C is Required for Transcriptional Activation of Estrogen/ER*α*‐Target Genes

**Figure 2:**
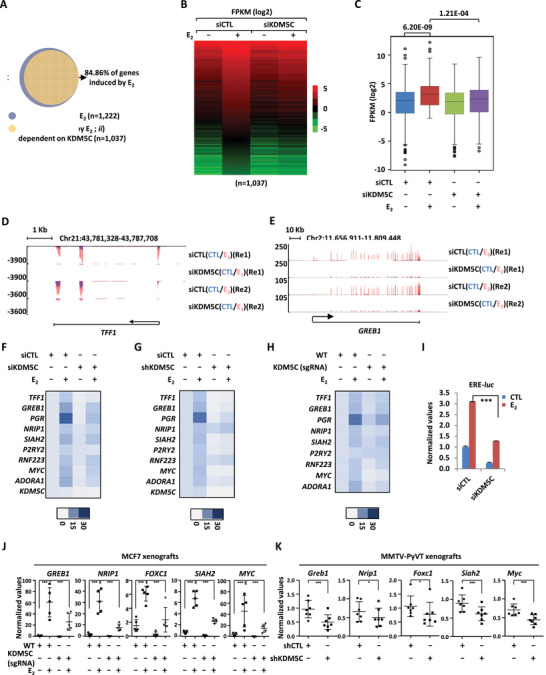
KDM5C is required for the activation of estrogen/ER*α*‐target genes. A) MCF7 cells were transfected with control siRNA (siCTL) or siRNA specific against KDM5C (siKDM5C) in stripping medium for three days, and then treated with or without estrogen (E_2_, 10^−7^ m, 6 h) followed by RNA‐seq. Genes induced by estrogen (E_2_) (fold change (*FC*) (siCTL (E_2_)/siCTL) ≥ 1.5) as well as those dependent on KDM5C for expression (fold change (*FC*) (siCTL (E_2_)/siKDM5C (E_2_)) ≥ 1.5) were shown. B,C) Heat map (B) and box plot (C) representation of the expression (FPKM, log2) for genes induced by estrogen (E_2_) and dependent on KDM5C as described in (A). D,E) Genome browser views of RNA‐seq as described in (A) for two estrogen/ER*α*‐target genes, *TFF1* (D) and *GREB1* (E), were shown. F) MCF7 cells were transfected with siCTL or siKDM5C in stripping medium for three days, and treated with or without estrogen (E_2_, 10^−7^ m, 6 h), followed by RNA extraction and RT‐qPCR analysis to examine the expression of selected estrogen‐induced genes as indicated. G) MCF7 cells were infected with shCTL or shKDM5C lenti‐virus in stripping medium for three days, and treated with or without estrogen (E_2_, 10^−7^ m, 6 h), followed by RNA extraction and RT‐qPCR analysis to examine the expression of selected estrogen‐induced genes as indicated. H) WT and KDM5C (sgRNA) MCF7 cells were maintained in stripping medium for three days, and treated with or without estrogen (E_2_, 10^−7^ m, 6 h), followed by RNA extraction and RT‐qPCR analysis to examine the expression of selected estrogen‐induced genes as indicated. I) HEK293T cells were transfected with siCTL or siKDM5C in the presence or absence of ER*α* and estrogen response element (ERE)‐driven luciferase reporter in stripping medium and treated with or without estrogen (E_2_, 10^−7^ m, 24 h) followed by luciferase reporter activity measurement (± SD., ^***^ *p* < 0.001). J,K) Tumor samples as described in Figure 1L (J) or Figure 1N (K) were subjected to RNA extraction and RT‐qPCR analysis to examine the expression of selected estrogen‐induced genes as indicated (± SD, ^*^ *p* < 0.05, ^***^ *p* < 0.001).

Prolonged exposure to high levels of the steroid hormone estrogen has been shown to be one of the major causes of ER‐positive breast cancers, in which the transcription of estrogen/ER*α*‐target genes, such as those with implications in cell cycle, metastasis, immune response, and metabolism, was constitutively activated. KDM5C's oncogenic role in ER*α*‐positive breast cancer prompted us to examine whether it is required for estrogen/ER*α*‐induced gene transcriptional activation. MCF7 cells were transfected with control siRNA or siRNA specifically targeting KDM5C, and then treated with or without estrogen, followed by RNA‐seq analysis. Around 1200 genes were strongly induced by estrogen treatment (*FC* > 1.5), and the expression of nearly 85% of these estrogen‐induced genes were attenuated when KDM5C was knocked down (*n =* 1037), which were referred to as estrogen‐induced and KDM5C‐dependent genes (**Figure** **2**A). The expression of these 1037 genes was shown by heat map (Figure 2B) and box plot (Figure 2C). KDM5C regulation of estrogen‐response was highly reproducible between two biological repeats of RNA‐seq (Figure S2A, Supporting Information). The expression of representative genes, such as *TFF1*, *GREB1*, *PGR*, and *NRIP1*, from RNA‐seq analysis was shown (Figure 2D,E; Figure S2B,C, Supporting Information). KDM5C's effects were validated by RT‐qPCR analysis on specific genes using two independent siRNAs targeting KDM5C in MCF7 cells (Figure 2F; Figure S2D–F, Supporting Information). shRNAs (Figure 2G; Figure S2G–I, Supporting Information) and sgRNAs‐mediated KDM5C KD (Figure 2H; Figure S2J–L, Supporting Information) exhibited similar effects. Furthermore, KDM5C's effects on representative estrogen‐target genes were demonstrated in another two ER*α*‐positive breast cancer cell line, T47D and BT474 (Figure S2M–P, Supporting Information). KDM5C's effects on estrogen/ER*α*‐mediated gene transcriptional activation might be due to its direct interaction with ER*α* as it was required for ERE‐driven luciferase reporter activation (Figure 2I and vide infra). To support the functional importance of these estrogen‐induced, KDM5C‐dependent genes in tumorigenesis, the expression of representative ones, such as *GREB1*, *NRIP1*, *FOXC1*, *SIAH2*, and *MYC*, was found to be significantly attenuated when KDM5C was knocked down in both MCF7 and MMTV‐PyVT xenografts (Figure 2J,K). Taken together, our data suggested that KDM5C activates estrogen‐target genes to promote breast cancer cell growth and tumorigenesis.

### 2.3 KDM5C Represses Type I Interferons and Interferon‐Stimulating Genes

**Figure 3:**
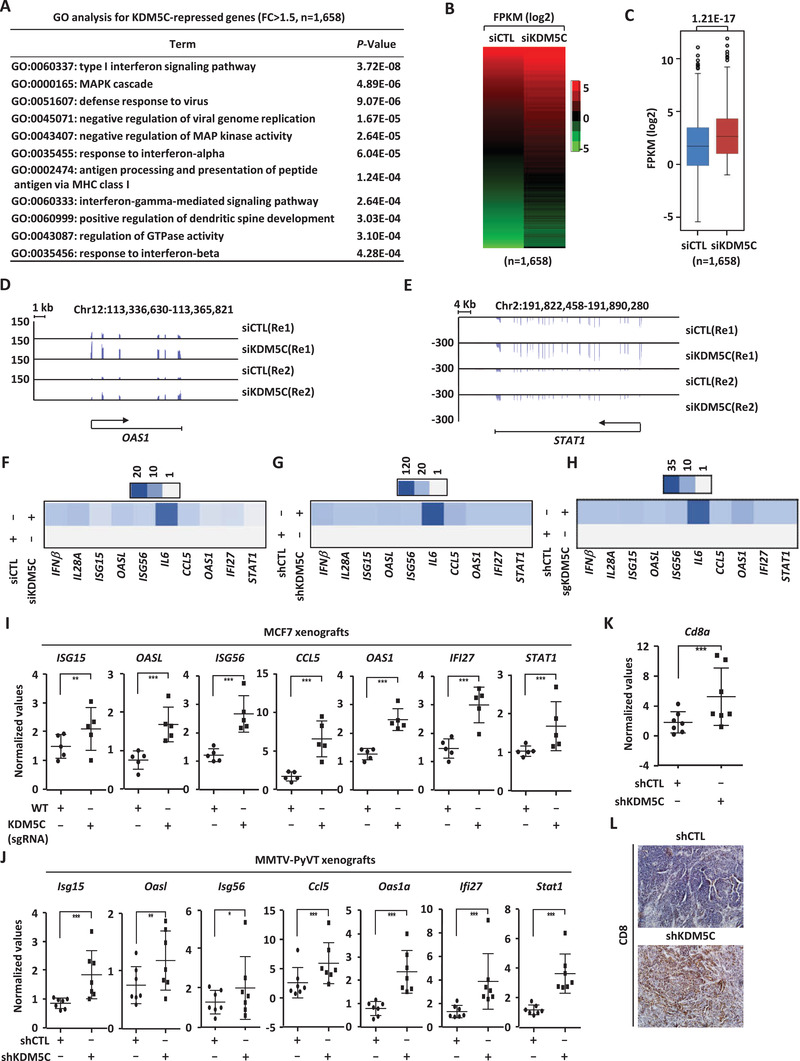
KDM5C represses type I interferons (IFNs) and interferon‐stimulating genes (ISGs). A) GO (gene ontology) analysis for genes repressed by KDM5C in MCF7 cells (*n =* 1658) (fold change (*FC*) (siKDM5C/siCTL) ≥ 1.5). Top enriched GO terms were shown. B,C) Heat map (B) and box plot (C) representation of the expression (FPKM, log2) for genes repressed by KDM5C (*n =* 1658). D,E) Genome browser views of representative genes repressed by KDM5C, such as OAS1 (D) and STAT1 (E), were shown. F) MCF7 cells were transfected with siCTL or siKDM5C for three days, followed by RNA extraction and RT‐qPCR analysis to examine the expression of selected type I IFNs and ISGs as indicated. G) MCF7 cells were infected with shCTL or shKDM5C lenti‐virus for three days, followed by RNA extraction and RT‐qPCR analysis to examine the expression of selected type I IFNs and ISGs as indicated. H) Wild type (WT) and KDM5C knockdown (KDM5C (sgRNA)) MCF7 cells were subjected to RNA extraction and RT‐qPCR analysis to examine the expression of selected type I IFNs and ISGs as indicated. I,J) Tumor samples as described in Figure 1L (I) or Figure 1N (J) were subjected to RNA extraction and RT‐qPCR analysis to examine the expression of selected type I IFNs and ISGs as indicated (± SD, ^*^ *p* < 0.05, ^**^ *p* < 0.01, ^***^ *p* < 0.001). K,L) Tumor samples as described in Figure 1N were subjected to RNA extraction and RT‐qPCR analysis (K) or immunohistochemistry (L) to examine the expression of Cd8a (± SD, ^***^ *p* < 0.001).

Besides activating estrogen‐induced gene program, KDM5C was found to repress the expression of 1658 genes in MCF7 cells when comparing siKDM5C‐transfected cells to control cells (*FC* > 1.5), among which genes with implications in type I IFN signaling pathway were the most enriched (**Figure** **3**A). The expression of these 1658 genes was shown by heat map (Figure 3B) and box plot (Figure 3C). Repression of gene transcription by KDM5C was highly reproducible between two biological repeats of RNA‐seq (Figure S3A, Supporting Information). The expression of representative genes, such as *OAS1* and *STAT1*, from RNA‐seq analysis was shown (Figure 3D,E). KDM5C's repressive effects on representative type I IFNs and ISGs were confirmed by RT‐qPCR analysis when KDM5C was knocked down by using two independent siRNAs, shRNAs, or sgRNAs (Figure 3F–H; Figure S3B–J, Supporting Information). Similar observations were made in T47D and BT474 cells (Figure S3K–N, Supporting Information). Restraining type I IFNs and type I ISGs from expression has been considered as a common mechanism for cancer cells to escape from immune surveillance.^[^ ^43^ ^]^ We therefore propose that suppression of these genes by KDM5C might, at least partially, account for its oncogenic role in ER*α*‐positive breast cancers. To support this notion, type I IFNs and ISGs, such as *ISG15*, *OASL*, *ISG56*, *CCL5*, *OAS1*, *IFI27*, and *STAT1*, were found to be upregulated when KDM5C was knocked down in both MCF7‐ and MMTV‐PyVT‐derived xenografts (Figure 3I,J). In MMTV‐PyVT‐derived xenografts in immunocompetent mice, upregulation of type I IFNs and ISGs was associated with increase of infiltrated CD8 T cells when KDM5C was knocked down (Figure 3K,L). Taken together, our data demonstrated that KDM5C can activate estrogen/ER*α*‐target genes to promote cell growth as well as repress type I IFNs and ISGs to escape from immune surveillance, both of which might contribute to its oncogenic function in ER*α*‐positive breast cancers.

### 2.4 KDM5C Activates Estrogen/ER*α*‐Target Genes Directly Through its Binding on ER*α*‐Bound Active Enhancers, but Represses Type I Interferons and Interferon‐Stimulating Genes Indirectly

**Figure 4:**
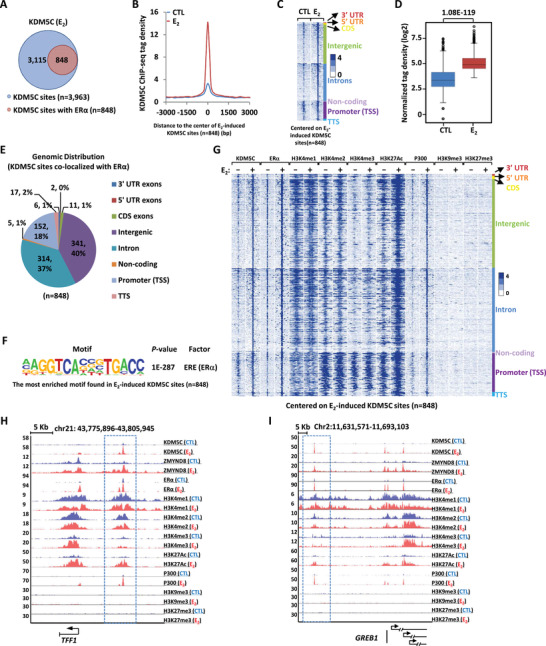
KDM5C activates estrogen/ER*α*‐target genes directly through its binding on ER*α*‐bound active enhancers. A) MCF7 cells treated with or without estrogen (E_2_, 10^−7^ m, 1 h) were subjected to ChIP‐seq with anti‐KDM5C specific antibody. KDM5C binding sites that overlapped with ER*α* in the presence of estrogen were shown as indicated. B) KDM5C ChIP‐seq tag density distribution, with or without estrogen (E_2_), centered on KDM5C sites that co‐localized with ER*α* (± 3000 bp). C,D) Heat map (C) and box plot (D) representation of the KDM5C ChIP‐seq tag density as shown in (B). E,F) Genomic distribution (E) and motif analysis (F) of KDM5C sites that co‐localized with ER*α*. G) Heat map representation of KDM5C, ER*α*, H3K4me1, H3K4me2, H3K4me3, H3K27Ac, P300, H3K9me3, and H3K27me3 ChIP‐seq tag density in the presence or absence of estrogen centered on KDM5C sites that co‐localized with ER*α* (± 3000 bp). H,I) UCSC Genome browser views of KDM5C, ZMYND8, ER*α*, H3K4me1, H3K4me2, H3K4me3, H3K27Ac, P300, H3K9me3, and H3K27me3 ChIP‐seq in the presence or absence of estrogen on selected active enhancer regions in the vicinity of estrogen‐induced target genes *TFF1* (H) and *GREB1* (I). Boxed regions indicated active enhancers.

To examine how KDM5C activates estrogen/ER*α*‐target genes, KDM5C ChIP‐seq was performed in MCF7 cells treated with or without estrogen using anti‐KDM5C specific antibody. It was found that, upon estrogen treatment, there were 848 KDM5C binding sites that were found to be strongly and significantly induced by estrogen, and co‐localized with ER*α* (**Figure** **4**A,G). The induction of KDM5C binding by estrogen was shown by tag density plot, heat map, and box plot analysis (Figure 4B–D). More than 80% of these estrogen‐induced KDM5C binding sites were localized at distal regions (non‐promoter regions) (Figure 4E). To understand the characteristics of these estrogen‐induced KDM5C binding sites, we performed motif analysis and found that ER*α* binding motif, ERE, was the most significantly enriched (Figure 4F). Enhancer characteristics, namely highly enriched H3K4me1/2, but low levels of H3K4me3, were evident from heat map and tag density plot for these estrogen‐induced KDM5C distal sites (Figure 4G; Figure S4A, Supporting Information). Furthermore, these estrogen‐induced KDM5C enhancer sites were enriched with activation markers including H3K27Ac and P300 (Figure 4G; Figure S4A, Supporting Information), but were devoid of repressive markers including H3K9me3 or H3K27me3 (Figure 4G; Figure S4A, Supporting Information). More importantly, the most robust ER*α* and activation markers (H3K27Ac and P300) were observed on these estrogen‐induced KDM5C enhancer sites (Figure S4A, Supporting Information, comparing the right panels to the left ones), which represented the group of active enhancers reported to be essential for the cognate, estrogen‐induced coding gene transcriptional activation.^[^ ^44^ ^]^ We then integrated estrogen‐induced KDM5C enhancer sites with estrogen‐induced, KDM5C‐dependent genes, and found that around 90% of estrogen‐induced, KDM5C‐dependent genes had KDM5C binding in vicinity, suggesting that KDM5C binding was required for the activation of majority of estrogen‐induced, KDM5C‐dependent genes. Occupancy of KDM5C, ER*α*, H3K4me1/2/3, H3K27Ac, P300, H3K9me3, and H3K27me3 on representative active enhancers were shown, such as the ones in the vicinity of estrogen‐induced coding genes *TFF1*, *GREB1*, *SIAH2*, and *P2RY2* (Figure 4H,I; Figure S4B,C, Supporting Information).

We next examined whether KDM5C binds to the promoter or adjacent enhancer regions of those type IFNs and ISGs it represses, and found no evidence of KDM5C binding, suggesting KDM5C might repress these genes indirectly. Taken together, KDM5C activates estrogen/ER*α*‐target genes directly through binding with ER*α*‐occupied active enhancers in vicinity, while represses type I IFNs and ISGs indirectly.

### 2.5 ZMYND8 Interacts with KDM5C and is Involved in Both KDM5C‐Activated and ‐Repressed Gene Expression

**Figure 5:**
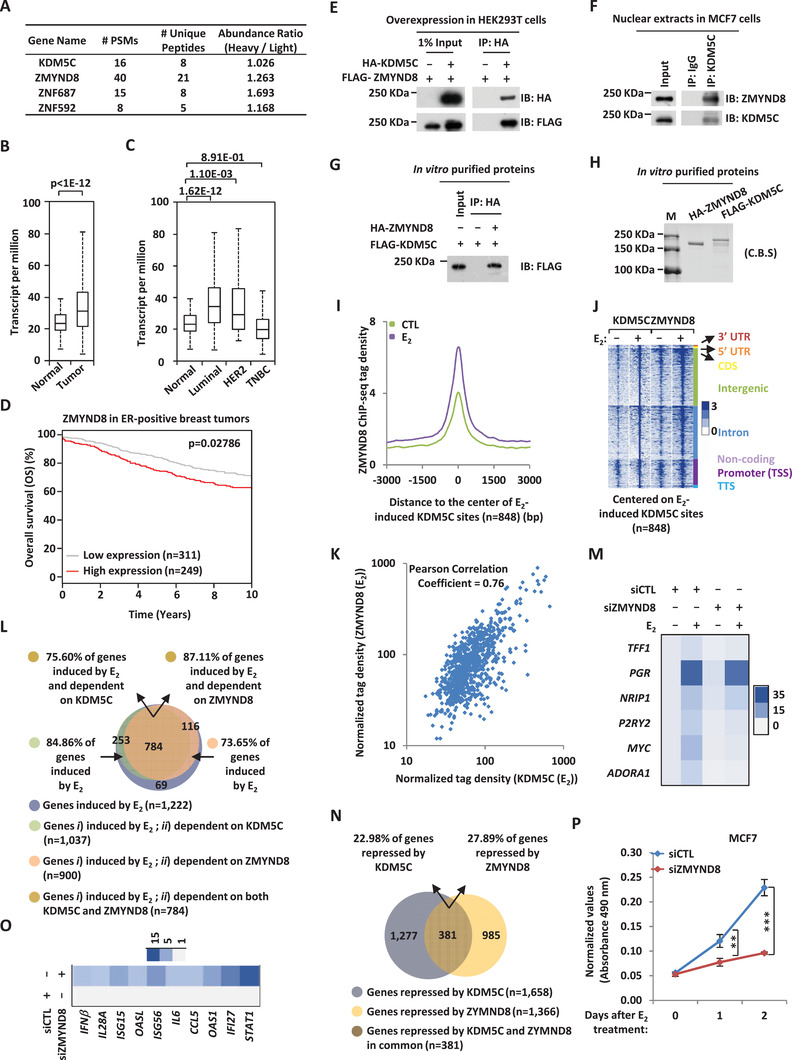
ZMYND8 interacts with KDM5C and is involved in the regulation of both KDM5C‐activated and ‐repressed genes. A) MCF7 cells stably expressing Flag‐HA‐tagged KDM5C were subjected to SILAC labeling and then treated with or without estrogen followed by cellular fractionation. Both nuclear and cytosolic fractions were subjected to affinity purification and mass spectrometry (MS) analysis. The number of peptide spectrum matches (PSMs), unique peptides, and the abundance ratio (estrogen vs control) for each subunit in the Z3 complex identified to interact with KDM5C in the nucleus was shown as indicated. B) The expression of ZMYND8 in a cohort of normal (*n =* 114) and clinical breast cancer (*n =* 1097) samples from TCGA (The Cancer Genome Atlas). C) The expression of ZMYND8 in different subtypes of clinical breast cancer samples from TCGA. (Normal, *n =* 114; Luminal, *n =* 566; HER2, *n =* 37; TNBC (triple‐negative breast cancer), *n =* 116). D) Kaplan–Meier survival analyses for OS (overall survival) of ER‐positive breast cancer patients using ZMYND8 as input (*n =* 560). E) Nuclear extracts from HEK293T cells transfected with HA‐tagged KDM5C and Flag‐tagged ZMYND8 were subjected to immunoprecipitation (IP) with anti‐HA antibody followed by immunoblotting (IB) analysis as indicated. F) Nuclear extracts from MCF7 cells were subjected to immunoprecipitation (IP) with control IgG or anti‐KDM5C antibody followed by immunoblotting (IB) analysis as indicated. G) In vitro pull‐down assay was performed by mixing in vitro purified HA‐tagged ZMYND8 and Flag‐tagged KDM5C proteins from HEK293T cells overexpressing these proteins, followed by immunoprecipitation (IP) with anti‐HA antibody and immunoblotting (IB) analysis with anti‐Flag antibody. H) In vitro purified HA‐tagged ZMYND8 and Flag‐tagged KDM5C proteins as described in (G) were examined by coomassie blue staining (C.B.S). I) MCF7 cells treated with or without estrogen (E_2_, 10^−7^ m, 1 h) were subjected to ChIP‐seq with anti‐ZYMND8 specific antibody, and ZYMND8 ChIP‐seq tag density centered on estrogen‐induced KDM5C binding sites was shown (± 3000 bp). J) Heat map representation of KDM5C and ZMYND8 ChIP‐seq tag density in the presence or absence of estrogen (E_2_) on estrogen‐induced KDM5C binding sites (± 3000 bp). K) Correlation between the ChIP‐seq tag density of KDM5C and ZMYND8 in the presence of estrogen (E_2_) on estrogen‐induced KDM5C binding sites. L) MCF7 cells transfected with control siRNA (siCTL) or siRNA specifically against KDM5C (siKDM5C) or ZMYND8 (siZYMND8) were treated with or without estrogen (E_2_, 10^−7^ m, 6 h) followed by RNA‐seq analysis. Estrogen‐induced genes (fold change (*FC*) (siCTL (E_2_)/siCTL) ≥ 1.5) which were dependent on KDM5C or ZMYND8 (fold change (*FC*) (siCTL (E_2_)/siKDM5C (E_2_) or siZMYND8 (E_2_)) ≥ 1.5) were shown by Venn diagram. M) RNA samples as described in (L) were subjected to RT‐qPCR analysis to examine the expression of selected estrogen‐induced genes as indicated. N) Overlapping between KDM5C‐ and ZYMND8‐repressed genes (fold change (*FC*) (siKDM5C or siZYMND8/siCTL) ≥ 1.5) was shown by Venn diagram O) Selected genes that were repressed by ZYMND8 were validated by RT‐qPCR analysis as indicated. P) MCF7 cells were transfected with control siCTL or siZYMND8 in stripping medium for three days, and then treated with or without estrogen (E_2_, 10^−7^ m) for different duration as indicated followed by cell proliferation assay (± SD, ^**^ *p* < 0.01, ^***^ *p* < 0.001).

In order to further understand the molecular mechanisms underlying KDM5C regulation of gene transcription, we sought to purify KDM5C‐associated proteins in MCF7 cells expressing Flag‐ and HA‐tagged KDM5C by affinity purification followed by mass spectrometry analysis. KDM5C was found to be localized in both nuclear and cytosolic fractions in MCF7 cells (Figure S5A, Supporting Information), and we therefore purified proteins associated with KDM5C both in the nucleus and cytosol by using nuclear and cytosolic extracts, respectively (Table S1, Supporting Information). We first analyzed the proteins associated with KDM5C in the nucleus in order to gain insights into the molecular mechanisms underlying direct regulation of estrogen/ER*α*‐target genes by KDM5C. Among all the proteins found to be associated with KDM5C, the “Z3 complex” (ZMYND8, ZNF687, and ZNF592), whose interaction with KDM5C was not changed significantly by estrogen treatment, was of particular interest (**Figure** **5**A; Table S1, Supporting Information). The Z3 complex, represented by ZMYND8, has been shown to interact with members in the KDM5 subfamily, such as KDM5C and KDM5D, to activate or repress gene expression.^[^ ^20^, ^35^, ^45^ ^]^ Similar as KDM5C, the expression of ZMYND8 was found to be significantly higher in clinical breast tumor samples than normal breast tissues (Figure 5B). Furthermore, ZMYND8 appeared to have the highest expression in luminal (ER‐positive) subtypes (Figure 5C). High expression of ZMYND8 was correlated with poor prognosis in ER‐positive breast cancer patients (Figure 5D).

To test whether ZMYND8 is involved in estrogen‐induced gene transcriptional activation, we first validated the interaction between ZMYND8 and KDM5C at both exogenous and endogenous level by using nuclear extracts (Figure 5E,F). In vitro pull‐down assay by using in vitro purified KDM5C and ZMYND8 proteins further demonstrated that the interaction between KDM5C and ZMYND8 was direct (Figure 5G,H). We then performed ZMYND8 ChIP‐seq in MCF7 cells treated with or without estrogen, and found that ZMYND8 binding was specifically and significantly induced on those KDM5C and ER*α* co‐bound active enhancer sites (Figure 5I,J). The binding intensity between ZMYND8 and KDM5C on these sites was highly correlated (Figure 5K).

We next sought to determine the sequence of recruitment of ER*α*, KDM5C, ZMYND8, and P300 onto enhancers. It has been reported previously that ZMYND8 is required for the recruitment of KDM5C to chromatin, but not vice versa.^[^ ^20^ ^]^ The dependency of KDM5C on ZMYND8 for binding on ER*α*‐bound active enhancers was shown (Figure S5B,C, Supporting Information). As ER*α* recruitment occurred in the first place during estrogen‐induced gene transcriptional activation, we then tested whether ER*α* is required for the recruitment of ZMYND8. MCF7 cells were treated with estrogen in the presence or absence of fulvestrant (ICI 182780, an ER*α* degrader) followed by ZMYND8 ChIP. It was found that the binding of ZMYND8 on ER*α*‐bound active enhancers was significantly decreased, suggesting that the binding of ZMYND8 was dependent on ER*α* (Figure S5D,E, Supporting Information). We also tested whether P300 interacts with KDM5C/ZMYND8, and if so, whether the binding between P300 and KDM5C/ZMYND8 is mutually dependent. The interaction between P300 and KDM5C/ZMYND8 was first demonstrated by immunoprecipitation (Figure S5F, Supporting Information). KD of P300 seemed to have no significant effects on or even slightly increased the binding of ZMYND8 and KDM5C on ER*α*‐bound active enhancers (data not shown). Similar experiments were performed to examine whether the binding of P300 is dependent on ZMYND8 and KDM5C, revealing that KD of ZMYND8 or KDM5C significantly attenuated the binding of P300 (Figure S5G–J, Supporting Information). Taken together, our data suggested that, upon estrogen treatment, ER*α* recruits ZMYND8/KDM5C, which further brings P300 onto ER*α*‐bound enhancers to deposit H3K27Ac to activate the enhancers.

ZMYND8 co‐localization with KDM5C on ER*α*‐bound active enhancers suggested that it might also be required for cognate estrogen‐induced gene transcriptional activation. To test this, MCF7 cells were transfected with control siRNA or siRNA specifically against KDM5C or ZMYND8 and then treated with or without estrogen before RNA‐seq analysis. The expression of around 85% and 74% of estrogen‐induced genes was attenuated following KDM5C and ZMYND8 KD, respectively, and the vast majority of KDM5C and ZMYND8‐affected genes were overlapped (Figure 5L). The impact of KDM5C and ZMYND8 on the expression of their commonly regulated genes was shown by heat map and box plot (Figure S6A,B, Supporting Information). ZMYND8 regulation of representative estrogen‐induced genes was confirmed by RT‐qPCR analysis using two independent siRNAs targeting ZMYND8 in MCF7 cells (Figure 5M; Figure S6C–G, Supporting Information). Similar observations were also made in T47D and BT474 cells (Figure S6H–K, Supporting Information). Our observation that ZMYND8 is required for KDM5C binding on ER*α*‐bound enhancers suggests that ZMYND8 is involved in KDM5C‐mediated estrogen/ER*α*‐target gene activation. To support this, double KD of ZMYND8 and KDM5C did not exhibit a synergistic effect (combination index, *CI* > 1) on the expression of estrogen‐target genes (Figure S6L,M, Supporting Information). The additional effects observed for KDM5C KD might due to the residual ZMYND8 proteins upon siRNA‐mediated KD.

We also examined ZMYND8's effects on the expression of those genes repressed by KDM5C by comparing siZMYND8‐transfected cells to control cells, and found that a large number of genes were commonly repressed by ZMYND8 and KDM5C (Figure 5N). The impact of KDM5C and ZMYND8 on the expression of their commonly repressed genes was shown by heat map and box plot (Figure S7A,B, Supporting Information). The repressive effect of ZMYND8 on representative genes, particularly those type I IFNs and ISGs, was confirmed by RT‐qPCR analysis using two independent siRNAs targeting ZMYND8 in MCF7 cells (Figure 5O; Figure S7C–E, Supporting Information). Similar observations were made in T47D cells (Figure S7F,G, Supporting Information). To strengthen the functional connection of ZMYND8 and KDM5C in gene transcriptional regulation, ZMYND8 was found to be required for ER*α*‐positive breast cancer cell growth similarly as KDM5C (Figure 5P; Figure S8A–C, Supporting Information). Taken together, our data suggested that ZMYND8 and KDM5C co‐regulate gene transcription, both activation and repression events.

### 2.6 ER*α* Interacts with KDM5C and Masks KDM5C's Demethylase Activity, Converting KDM5C from a Transcriptional Repressor to Activator During Activation of Estrogen/ER*α*‐Target Genes

**Figure 6:**
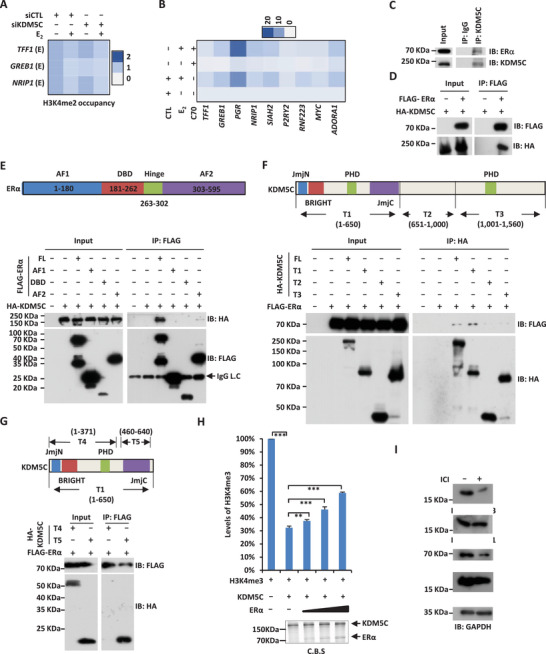
KDM5C activates estrogen/ER*α*‐target genes in an enzymatic‐independent manner due to its interaction with ER*α*. A) MCF7 cells were transfected with control siRNA (siCTL) or siRNA specific against KDM5C (siKDM5C) in stripping medium for three days, and treated with or without estrogen (E_2_, 10^−7^ m, 1 h) followed by H3K4me2 ChIP analysis. The occupancy of H3K4me2 was examined for selected active enhancer regions in the vicinity of estrogen‐induced genes as indicated. B) MCF7 cells were maintained in stripping medium for three days, and pre‐treated with or without C70 (5 *μ* m, 3 days) before treating with or without estrogen (E_2_, 10^−7^ m, 6 h) followed by RNA extraction and RT‐qPCR analysis to examine the expression of selected estrogen‐induced genes as indicated. C) MCF7 cells treated with estrogen (E_2_, 10^−7^ m, 1 h) were subjected to immunoprecipitation (IP) with anti‐KDM5C antibody followed by immunoblotting (IB) analysis as indicated. D) HEK293T cells transfected with HA‐tagged KDM5C and Flag‐tagged ER*α* with a nuclear localization signal were subjected to immunoprecipitation (IP) with anti‐Flag antibody followed by immunoblotting (IB) analysis as indicated. E) HEK293T cells transfected with HA‐tagged KDM5C and Flag‐tagged, full length (FL) or truncated forms of ER*α* were subjected to immunoprecipitation (IP) with anti‐Flag antibody followed by immunoblotting (IB) analysis as indicated. AF1: Activation function domain 1; AF2: Activation function domain 2; DBD: DNA binding domain; IgG L.C; IgG light chain. F) HEK293T cells transfected with Flag‐tagged ER*α* and HA‐tagged, full length (FL) or truncated forms of KDM5C were subjected to immunoprecipitation (IP) with anti‐HA antibody followed by immunoblotting (IB) analysis as indicated. T1: aa (1–650); T2: aa (651–1000); T3: aa (1001–1560). G) HEK293T cells transfected with Flag‐tagged ER*α* and truncated forms of amino‐terminal of KDM5C were subjected to immunoprecipitation (IP) with anti‐HA antibody followed by immunoblotting (IB) analysis as indicated. T4: aa (1–371); T5: aa (460–640). H) KDM5C demethylase activity was quantified by counting the percentage of H3K4me3 peptides as shown in Figure S9I, Supporting Information (± SD, ^**^ *p* < 0.01, ^***^ *p* < 0.001). The amount of KDM5C and ER*α* proteins loaded were examined by coomassie blue staining (C.B.S). I) MCF7 cells were treated with or without fulvestrant (ICI 182780, ICI, 1 *μ* m, 3 days) followed by immunoblotting (IB) analysis with antibodies as indicated.

The unexpected role of KDM5C in activating ER*α*‐target genes promoted us to examine what triggers the conversion of KDM5C from a co‐repressor to co‐activator. The repressive function of KDM5C in gene transcriptional regulation was primarily through its demethylase activity targeting H3K4me2/3, an activation marker. We therefore first test whether it loses its H3K4me2/3 demethylase activity on active enhancers where it binds. MCF7 cells transfected with control siRNA or siRNA specific against KDM5C were treated with or without estrogen followed by ChIP analysis with anti‐H3K4me2 antibody. KD of KDM5C did not lead to a significant increase of H3K4me2 occupancy on any of the KDM5C‐bound ER*α* enhancer regions tested, such as those in the vicinity of *TFF1*, *GREB1*, and *NRIP1* genes, either in the presence or absence of estrogen, indicating that KDM5C lost its demethylase activity targeting H3K4me2 (**Figure** **6**A; Figure S9A, Supporting Information). Similarly, KD of KDM5C had no significant impact on H3K4me3 intensity on these enhancer regions (Figure S9B,C, Supporting Information). In contrast, KD of KDM5C attenuated estrogen‐induced H3K4me3 occupancy on the promoter regions of estrogen/ER*α*‐induced target genes, such as *TFF1*, which was consistent with the fact that KDM5C was required for the transcriptional activation of these genes (Figure S9D, Supporting Information). Loss of demethylase activity of KDM5C appeared to be local on ER*α*‐bound active enhancer regions as KD of KDM5C still caused a global increase of H3K4me3 levels as examined by immunoblotting (Figure S9E, Supporting Information). To further strengthen the notion that KDM5C was inactive during estrogen‐induced gene transcriptional activation, C70, a KDM5 inhibitor,^[^ ^46^ ^]^ exhibited no significant impact on the expression of estrogen/ER*α*‐target genes (Figure 6B; Figure S9F, Supporting Information). In certain cases, C70 even led to further induction of estrogen/ER*α*‐target genes, which might be due to indirect effects (Figure 6B). Rescue experiments with WT or catalytic mutant (H514A) KDM5C were also performed, revealing that KD of KDM5C impaired the expression of selective estrogen‐target genes, and both WT and catalytic inactive KDM5C appeared to rescue estrogen‐induced expression in a similar fashion, indicating that enzymatic activity of KDM5C is not required for the transcriptional activation of estrogen‐target genes (Figure S9G,H, Supporting Information).

We next sought to investigate what triggers KDM5C to lose its enzymatic activity during activation of estrogen/ER*α*‐target genes. It has been reported that, when Lid, the only Drosophila KDM5 ortholog, participates in dMyc‐induced expression of cell growth genes, its enzymatic activity is masked by dMyc, which binds to Lid's JmjC domain.^[^ ^28^ ^]^ The co‐localization of KDM5C and ER*α* on enhancers as well as KDM5C's ability to activate ERE‐driven luciferase reporter activity as described above suggested that KDM5C itself might bind to ER*α*, which could serve as a potential mechanism for the inactivation of KDM5C's enzymatic activity and changing from a co‐repressor to a co‐activator locally on ER*α*‐bound active enhancers. We first demonstrated the interaction between KDM5C and ER*α* at both endogenous and exogenous levels (**Figure** **6**C,D). Next, we mapped the domain in ER*α* to interact with KDM5C by co‐expressing full length or different truncated form of ER*α* with KDM5C followed by immunoprecipitation, finding that AF2 domain in ER*α* was interacting with KDM5C (Figure 6E). Similar approach was utilized to map the domain in KDM5C to interact with ER*α*, which was first determined to be the amino (N)‐terminal of KDM5C encompassing the JmjN, BRIGHT, PHD, and JmjC domains (Figure 6F), and further mapped to be the JmjC domain, which is the catalytic domain in KDM5C (Figure 6G).ER*α* interaction with the catalytic domain of KDM5C suggested that it might inhibit KDM5C enzymatic activity. To test this, in vitro demethylation assay was performed by mixing H3K4me3 peptide with KDM5C in the presence or absence of ER*α*. It was found that KDM5C was capable of converting H3K4me3 to both H3K4me2 and H3K4me1 efficiently, which was significantly attenuated by ER*α* in a dose‐dependent manner (Figure 6H; Figure S9I, Supporting Information). Downregulation of ER*α* by fulvestrant treatment led to a dramatic increase of H3K4me3, but not H3K4me1, further supporting that ER*α* might inhibit KDM5C's enzymatic activity in cells (Figure 6I). Taken together, our data suggested that ER*α* binds to KDM5C and masks its demethylase activity on those KDM5C and ER*α*‐bound active enhancers, converting KDM5C from a co‐repressor to a co‐activator during estrogen‐induced gene transcriptional activation.

### 2.7 KDM5C and ZMYND8 Interact with CDK9 and CCNT1 in the P‐TEFb Complex, Respectively, to Activate Estrogen/ER*α*‐Target Genes

**Figure 7:**
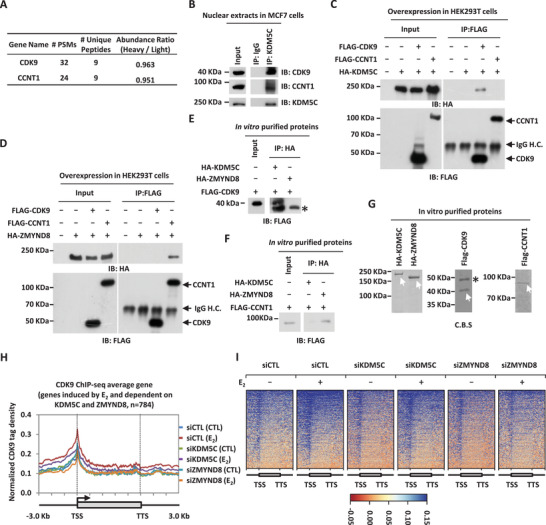
KDM5C and ZMYND8 interact with CDK9 and CCNT1 in the P‐TEFb complex, respectively, to activate estrogen/ER*α*‐target genes. A) The number of peptide spectrum matches (PSMs), unique peptides, and the abundance ratio (estrogen vs control) for CDK9 and CCNT1 proteins identified to be associated with KDM5C in the nucleus as described in Figure 5A was shown. B) Nuclear extracts from MCF7 cells were subjected to immunoprecipitation (IP) with anti‐KDM5C antibody followed by immunoblotting (IB) analysis as indicated. C) Nuclear extracts from HEK293T cells transfected with HA‐tagged KDM5C and Flag‐tagged CDK9 or CCNT1 were subjected to immunoprecipitation (IP) with anti‐Flag antibody followed by immunoblotting (IB) analysis as indicated. IgG H.C: IgG heavy chain. D) Nuclear extracts from HEK293T cells transfected with HA‐tagged ZYMND8 and Flag‐tagged CDK9 or CCNT1 were subjected to immunoprecipitation (IP) with anti‐Flag antibody followed by immunoblotting (IB) analysis as indicated. IgG H.C: IgG heavy chain. E) In vitro pull‐down assay was performed by mixing in vitro purified Flag‐tagged CDK9 and HA‐tagged KDM5C or ZYMND8 proteins from HEK293T cells overexpressing these proteins, followed by immunoprecipitation (IP) with anti‐HA antibody and immunoblotting (IB) analysis with anti‐Flag antibody. F) In vitro pull‐down assay was performed by mixing in vitro purified Flag‐tagged CCNT1 and HA‐tagged KDM5C or ZYMND8 proteins from HEK293T cells overexpressing these proteins, followed by immunoprecipitation (IP) with anti‐HA antibody and immunoblotting (IB) analysis with anti‐Flag antibody. G) In vitro purified HA‐tagged KDM5C and ZYMND8, and Flag‐tagged CDK9 and CCNT1 proteins were examined by coomassie blue staining (C.B.S) and indicated by white arrows. Non‐specific band was indicated by asterisk. H) MCF7 cells were transfected with control siRNA (siCTL) or siRNA specific against KDM5C (siKDM5C) or ZYMND8 (siZYMND8) in stripping medium for three days, and treated with or without estrogen (E_2_, 10^−7^ m, 1 h) followed by CDK9 ChIP‐seq analysis. Metagenes of CDK9 ChIP‐seq tag density across those genes induced by estrogen and dependent on KDM5C and ZYMND8 for expression (*n =* 784) were shown. Units are mean tags per bin for 500 bins across the transcribed region of each gene with 3 kb upstream (300 bins of 10 bp each) and 3 kb downstream flanking regions (300 bins of 10 bp each). I) Heat map representation of CDK9 ChIP‐seq tag density as shown in (H).

KDM5C's enzymatic activity was inactivated by ER*α*, but what enables KDM5C/ZMYND8 protein complex to activate estrogen‐induced gene transcriptional activation remains unclear. To this end, we re‐examined KDM5C‐interacting proteins in the nucleus revealed by affinity purification as described above, finding that the P‐TEFb complex, including CDK9 and CCNT1, was abundantly present (**Figure** **7**A; Table S1, Supporting Information). The interaction between KDM5C and the P‐TEFb complex was validated at endogenous protein level (Figure 7B). To further illustrate the interaction network of KDM5C, ZMYND8, CDK9 and CCNT1, we transfected cells with HA‐tagged KDM5C and Flag‐tagged CDK9 or CCNT1 followed by immunoprecipitation and immunoblotting, and found that KDM5C preferentially bound to CDK9 (Figure 7C). Similarly, we examined the interaction between ZMYND8 and CDK9 or CCNT1, and found that, intriguingly, ZMYND8 instead preferentially bound to CCNT1 (Figure 7D). The preferential interaction of KDM5C with CDK9, and ZMYND8 with CCNT1 was further validated in vitro (Figure 7E–G).

The intriguing interacting network, in which KDM5C and ZMYND8 preferentially bind to CDK9 and CCNT1, respectively, prompted us to test whether KDM5C and ZMYND8 are both required for the recruitment of the P‐TEFb complex, which further leads to the transcriptional activation of estrogen/ER*α* target genes. To this end, we transfected MCF7 cells with control siRNA or siRNA specifically against KDM5C or ZMYND8 and then treated with or without estrogen followed by ChIP‐seq for CDK9, which represents the P‐TEFb complex. As expected, estrogen treatment led to a dramatic increase of CDK9 occupancy on both promoter and gene body regions of those genes which were induced by estrogen and dependent on KDM5C and ZYMND8 for expression (Figure 7H,I). Estrogen‐induced CDK9 occupancy was significantly attenuated when KDM5C or ZMYND8 was knocked down as shown by tag density plot and heat map analysis (Figure 7H,I). Taken together, KDM5C and ZMYND8 interact and recruit the P‐TEFb complex to activate estrogen/ER*α*‐target genes.

### 2.8 KDM5C Inhibit TBK1 Phosphorylation to Repress Type I Interferons and Interferon‐Stimulating Genes

**Figure 8:**
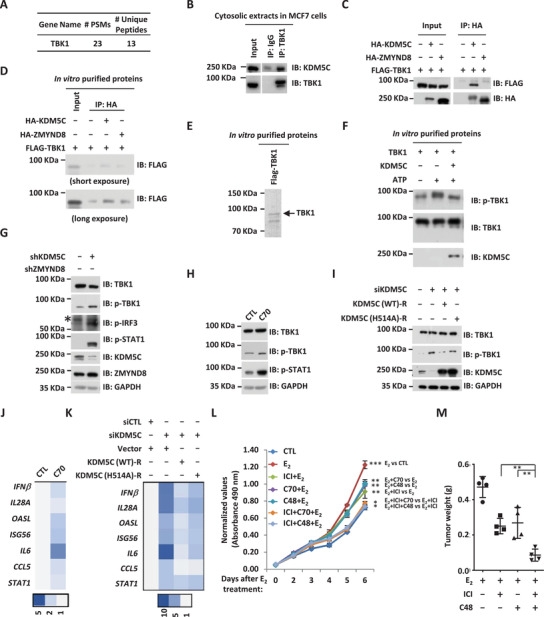
KDM5C interacts with TBK1 to inhibit its phosphorylation and the expression of type I IFNs and ISGs. A) The number of peptide spectrum matches (PSMs) and unique peptides for TBK1 protein identified to be associated with KDM5C in the cytosol as described in Figure 5A was shown. B) Cytosolic extracts from MCF7 cells were subjected to immunoprecipitation (IP) with anti‐TBK1 antibody followed by immunoblotting (IB) analysis as indicated. C) Cytosolic extracts from HEK293T cells transfected with Flag‐tagged TBK1 and HA‐tagged KDM5C or ZMYND8 were subjected to immunoprecipitation (IP) with anti‐HA antibody followed by immunoblotting (IB) analysis as indicated. D) In vitro pull‐down assay was performed by mixing in vitro purified Flag‐tagged TBK1 and HA‐tagged KDM5C or ZYMND8 proteins from HEK293T cells overexpressing these proteins, followed by immunoprecipitation (IP) with anti‐HA antibody and immunoblotting (IB) analysis with anti‐Flag antibody. E) In vitro purified Flag‐tagged TBK1 protein as described in (D) was examined by coomassie blue staining (C.B.S). F) In vitro phosphorylation assay was performed by mixing in vitro purified Flag‐tagged TBK1 and HA‐tagged KDM5C proteins from HEK293T cells overexpressing these proteins, followed by immunoblotting (IB) analysis with antibodies as indicated. p‐TBK1: Anti‐TBK1 phosphorylated at serine 172 antibody. G) MCF7 cells were infected with lenti‐viral control shRNA (shCTL) or shRNA specific against KDM5C (shKDM5C) or ZYMND8 (shZYMND8) for three days, followed by immunoblotting analysis using antibodies as indicated. p‐IRF3: Anti‐IRF3 phosphorylated at serine 396 antibody; p‐STAT1: Anti‐STAT1 phosphorylated at tyrosine 701 antibody. Asterisk indicated non‐specific band. H,J) MCF7 cells were treated with or without C70 (5 *μ* m, 3 days) followed by immunoblotting (IB) analysis using antibodies as indicated (H) or RT‐qPCR analysis to examine the expression of selected genes as indicated (J). Asterisk indicated non‐specific band. I,K) MCF7 cells were transfected with siCTL or siKDM5C in the presence or absence of control vector, KDM5C (WT)‐R or KDM5C (H514A)‐R for three days, and then treated with or without estrogen (E_2_, 10^−7^ m, 6 h) followed by immunoblotting (IB) analysis using antibodies as indicated (I) or RT‐qPCR analysis to examine the expression of selected genes as indicated (K). Asterisk indicated non‐specific band. L) MCF7 cells cultured in the presence of estrogen (E_2_, 10^−7^ m) were treated with or without fulvestrant (ICI, 1 *μ* m for MCF7, 0.1 *μ* m for T47D), C70 (5 *μ* m), or C48 (10 *μ* m) alone or in combination for duration as indicated followed by cell proliferation assay (± SD, ^*^ *p* < 0.05, ^**^ *p* < 0.01, and ^***^ *p* < 0.001). M) Xenograft experiments were performed by injecting MCF7 cells subcutaneously into female BALB/C nude mice and then treated with estrogen (E_2_) in the presence of fulvestrant (ICI) or C48 alone or in combination. Tumors were then excised, photographed, and weighted four weeks after subcutaneous injection (± SD, ^**^ *p* < 0.01).

We next sought to investigate how KDM5C represses gene transcription, particularly those type I IFNs and ISGs. Cancer cells have evolved multiple mechanisms to escape from immune surveillance, among which suppressing the expression of type I IFNs and ISGs has been well studied. Signaling cascades in the cytosol have been shown to be involved in the activation of type I IFNs and ISGs, such as cGAS‐STING induced TBK1 phosphorylation. A significant portion of KDM5C was found to be localized in the cytosol of cell (Figure S10A, Supporting Information). We therefore examine whether KDM5C could interact with any of the proteins in the signaling cascades leading to type I IFNs and ISGs activation by searching the proteins found to be associated with KDM5C in the cytosolic fractions. It was found that TBK1 was abundantly present in the list of proteins associated with KDM5C in the cytosol (**Figure** **8**A). We first validated the interaction between TBK1 and KDM5C at endogenous level (Figure 8B). To test whether KDM5C directly interacts with TBK1, we transfected HEK293T cells with vectors expressing TBK1 and KDM5C or ZMYND8 followed by immunoprecipitation and immunoblotting, finding that TBK1 preferentially interacted with KDM5C (Figure 8C). In vitro pull‐down assay using in vitro purified TBK1 and KDM5C or ZMYND8 confirmed the preferential interaction between TBK1 and KDM5C (Figure 8D,E).

The direct interaction between KDM5C and TBK1 prompted us to test whether KDM5C inhibits TBK1 phosphorylation, which would account for its repressive effects on type I IFNs and ISGs expression. To this end, we performed in vitro kinase assay by mixing in vitro purified TBK1 with or without KDM5C, finding that TBK1 auto‐phosphorylation was significantly attenuated in the presence of KDM5C, but not GST proteins (Figure 8F; Figure S10B, Supporting Information). In accordance with the observed inhibitory effects of KDM5C on TBK1 phosphorylation in vitro, KD of KDM5C led to a significant increase of TBK1 phosphorylation as well as the downstream signaling events, such as IRF3 and STAT1 phosphorylation (Figure 8G). KDM5C inhibition of TBK1 phosphorylation appeared to be dependent its enzymatic activity as C70 treatment also led to a significant increase of TBK1, IRF3, and STAT1 phosphorylation (Figure 8H). Furthermore, rescue experiments demonstrated that KDM5C (WT) largely diminished the increase of TBK1 phosphorylation caused by KDM5C KD, but catalytic inactive mutant did so in a much less extent, suggesting that KDM5C inhibits TBK1 phosphorylation in an enzymatic activity‐dependent manner (Figure 8I). Consequently, C70 treatment led to de‐repression of type I IFNs and ISGs (Figure 8J), and KDM5C (WT) largely diminished the upregulation of type I IFNs and ISGs caused by KDM5C KD, but catalytic inactive mutant did so in a much less extent, suggesting repression of these genes by KDM5C was dependent on its enzymatic activity (Figure 8K; Figure S10C, Supporting Information). Taken together, our data suggested that KDM5C represses type I IFNs and ISGs in an enzymatic‐dependent manner through direct interaction with TBK1 and inhibition of TBK1 phosphorylation.

### 2.9 Combination Treatment with ER*α* Antagonist and KDM5 Inhibitor is Effective in Suppressing ER*α*‐Positive Breast Cancer Cell and Tumor Growth

Our data above suggested that KDM5C activates estrogen/ER*α*‐target gene independent on its enzymatic activity, while represses type I IFNs and ISGs in an enzymatic‐dependent manner, with both are beneficial for breast cancer cell growth and tumorigenesis. We propose that simultaneously blocking estrogen/ER*α*‐mediated gene activation by ER*α* antagonist and activation of type I IFNs and ISGs expression by KDM5C inhibitor might be an effective way to suppress ER*α*‐positive breast cancer cell and tumor growth. To this end, MCF7 cells cultured in the presence or absence of estrogen were treated with fulvestrant or C70 alone or in combination followed by cell proliferation assay. Co‐treatment with both fulvestrant and C70 exhibited synergistic effects on cell growth compared to treatment with either fulvestrant or C70 alone (Figure 8L). Similar observations were made when cells were co‐treated with fulvestrant and another KDM5C inhibitor, C48^[^ ^47^ ^]^ (Figure 8L). The synergistic effects of ER*α* antagonist and KDM5C inhibitor were also seen in T47D and BT474 cells (Figure S11A,B, Supporting Information). We next tested the effects of co‐treatment in suppressing MCF7 xenograft tumor growth. In consistent with what observed in cultured cells, fulvestrant and C48 co‐treatment was more effective in suppressing tumor growth, and exhibited no apparent side effects on body weight (Figure 8M and data not shown). Taken together, our data suggested that simultaneously blocking ER*α* signaling by ER*α* antagonist and KDM5C activity by KDM5C inhibitor is an effective way to suppress ER*α*‐positive breast cancer cell and tumor growth.

## 3 Discussion

Epigenetic regulators often exhibit dual activities, both activation and repression, in gene transcriptional regulation, and disease development, such as cancer. However, how these dual activities are regulated and coordinated remain incompletely understood. Here, we focused on studying KDM5C, a JmjC domain‐containing demethylase, in ER*α*‐positive breast cancers, and found that KDM5C‐activated and ‐repressed gene programs were both implicated in cancer development, which were regulated by KDM5C via distinct mechanisms, such that KDM5C activates estrogen/ER*α*‐target genes directly through binding to ER*α*‐occupied active enhancers nearby in an enzymatic‐independent manner in the nucleus, while represses type I IFNs and ISGs indirectly through modulating TBK1 phosphorylation in an enzymatic‐dependent manner in the cytosol (Figure S12, Supporting Information).

Numerous studies have suggested that epigenetic regulators, such as histone demethylase family, are involved in cancer development. We systematically screened the JmjC domain‐containing and LSD demethylase family proteins to identify candidates that are required for estrogen‐induced growth of ER*α*‐positive breast cancer cells. In accordance with previous reports, many of the demethylases were found to be required for estrogen‐induced cell growth.^[^ ^42^ ^]^ In the current story, we focused on studying KDM5C's functions and the underlying molecular mechanisms due to its high expression and the associated poor prognosis in ER‐positive breast cancer patients. The aberrant activation of estrogen/ER‐target gene program was considered as one of the main driving‐forces for ER‐positive breast cancer development as this gene program is implicated in cell cycle regulation, cell metabolism control, and immune response, among others. Transcriptomics analysis revealed that, unexpectedly, KDM5C was required for the transcriptional activation of the vast majority of estrogen‐induced target genes. Meanwhile, KDM5C was found to repress a large cohort of genes, among which the type I IFNs and ISGs were the most enriched, suggesting that KDM5C might protect cancer cells from immune surveillance by restraining these genes from expression. Several epigenetic regulators, such as LSD1, SETD2, and DNMTs were reported to suppress the expression of type I IFNs and ISGs and therefore tumor immunogenicity, suggesting that this might represent a common mechanism utilized by cancer cells.^[^ ^48^ ^]^ The dual activities of KDM5C, activating estrogen‐target genes and repressing type I IFNs and ISGs, were confirmed in xenograft mouse models, which might together contribute to its oncogenic nature in ER*α*‐positive breast cancers.

An outstanding question regarding the dual nature of epigenetic regulators in gene transcriptional regulation is that how these dual activities are regulated and coordinated in cellular contexts. Genome‐wide mapping of KDM5C binding sites revealed distinct spatial regulatory modes for KDM5C‐activated and ‐repressed genes, with KDM5C being recruited to ER*α*‐bound active enhancers in response to estrogen treatment to directly activate estrogen/ER*α*‐target genes, while no evident binding of KDM5C was observed for the repressed type I IFNs and ISGs.

The activation function of KDM5C is obviously contradictory to its H3K4me2/3 demethylase activity, which prompted us to examine whether KDM5C loses its enzymatic activity during estrogen‐induced gene transcriptional activation, and if so, what triggers the silencing of KDM5C enzymatic activity. Several lines of evidence indicated that ER*α* might interact with KDM5C directly, serving as the trigger to silent KDM5C demethylase activity: i) KDM5C binding was highly correlated with that of ER*α* on estrogen‐induced KDM5C sites; ii) KDM5C was required for the activation of ERE‐driven luciferase reporter activity; iii) ER*α* was present in the list of proteins found to be associated with KDM5C when we purified KDM5C‐interacting proteins. Through a series of biochemical characterization, ER*α* interacted with KDM5C's catalytic domain, which was proven to mask and silent KDM5C enzymatic activity. The co‐activator function has been reported for other members in the KDM5 protein family in nuclear receptor‐mediated gene transcriptional activation. For instance, KDM5A was reported to potentiate ER‐mediated transcription, and KDM5B did so for both ER and AR (androgen receptor), though whether the demethylase activity was lost for KDM5A and KDM5B remained uncharacterized.^[^ ^9^, ^21^, ^49^ ^]^

Masking KDM5C's demethylase activity by ER*α* did not fully explain its unexpected role in gene transcriptional activation. We therefore purified KDM5C‐interacting proteins and found that KDM5C interacted with several protein complexes including the “Z3 complex” (ZMYND8, ZNF687, and ZNF592),^[^ ^45^ ^]^ the NuRD complex,^[^ ^45^ ^]^ the SWI/SNF complex, the P‐TEFb complex, transcription factor II D complex, transcription factor II H complex, mediator complex, PRC2 complex, SIN3A corepressor complex, nucleotide excision repair complex, and non‐homologous end‐joining complex, among others. We focused on studying the interaction between KDM5C and ZYMND8 and their contributions to estrogen‐induced gene transcriptional activation due to the following reasons: i) The role of KDM5C and ZYMND8 in breast cancers has been controversial. It has been reported that KDM5C/ZYMND8 prevent the hyperactivation of super enhancers and thus the cognate oncogenes, such as S100A family genes, to inhibit tumorigenic capability of breast cancer cells,^[^ ^20^ ^]^ while several other studies described the opposite that KDM5C or ZMYND8 is oncogenic in breast cancers.^[^ ^18^, ^19^, ^39^, ^40^ ^]^ To support the oncogenic role of these two proteins, both KDM5C and ZYMND8's expression are inversely associated with survival in breast cancer patients.^[^ ^18^, ^19^, ^39^, ^40^ ^]^ Here, we found that KDM5C and ZMYND8 activated as well as repressed a common set of genes, both of which contributed to breast cancer cell growth. The controversy about KDM5C and ZMYND8's function in tumorigenesis might be due to the different role they play in different stages of tumor. For instance, they promote the growth of tumor cells at early stage and therefore are oncogenic, but suppress the invasion and metastasis at late stage and therefore are tumor suppressive. ii) ZYMND8 was reported to directly interact with the CCNT1 subunit in the P‐TEFb complex to activate ATRA‐induced neuronal gene expression, and KDM5C was found to pull down the P‐TEFb complex, both CDK9 and CCNT1, in our affinity purification. We therefore propose that there might be an interaction network between KDM5C, ZMYND8, and P‐TEFb, which could serve as a potential mechanism for KDM5C/ZMYND8‐mediated gene activation. Indeed, a unique network was revealed for these four proteins, in which KDM5C and ZYMND8 preferentially interact with CDK9 and CCNT1 in the P‐TEFb complex, respectively. Functionally, KD of KDM5C or ZYMND8 attenuated the occupancy of the P‐TEFb complex on estrogen/ER*α*‐target genes coactivated by KDM5C and ZYMND8, suggesting a double‐safelock‐mode in the recruitment of P‐TEFb complex to ensure gene activation.

Repression of gene expression including those type I IFNs and ISGs by KDM5C appeared to be indirect as no binding of KDM5C was observed on either these genes’ promoter or enhancer regions, which prompted us to test whether KDM5C is involved in the signaling pathways that lead to the activation of type I IFNs and ISGs. Indeed, KDM5C was found to interact with TBK1 in the cytosol and inhibited its phosphorylation, which was known to be a critical molecular event in activating type I IFNs and ISGs. Intriguingly, inhibition of TBK1 phosphorylation and type I IFNs and ISGs was found to be dependent on KDM5C enzymatic activity. However, why KDM5C enzymatic activity was crucial here remains unclear. One of the possible mechanisms could be that TBK1 itself might serve as a non‐histone substrate for KDM5C, and methylation on TBK1 is crucial for its activation. Recently, it was shown that KDM5C directly represses the expression of STING through removing the H3K4me2/3 markers on its promoter, leading to the inactivation of STING and repression of type I IFNs and ISGs.^[^ ^19^ ^]^ More recently, it was reported that loss of ZMYND8 increases micronucleus formation and activates the cGAS‐STING signaling cascade to induce IFN*β* and ISGs in breast cancer cells and tumors.^[^ ^41^ ^]^ Therefore, regulation of type I IFNs and ISGs by KDM5C and ZYMND8 seemed to be occurred at multiple checkpoints, and they might serve as gatekeepers to block the activation of type I IFNs and ISGs to escape from immune surveillance. It should be noted that other genes, such as some members in the S100A family, were also found to be repressed by KDM5C and ZYMND8, which was consistent with previous report.^[^ ^20^ ^]^

Our data thereby revealed that KDM5C can activate estrogen/ER*α*‐target genes directly in a demethylase activity‐independent manner, while repress type I IFNs and ISGs indirectly via its demethylase activity. Both KDM5C's activator and repressor nature contribute to its oncogenic function in ER‐positive breast cancer development, and therefore it might serve as a potential drug target in ER‐positive and endocrine therapy‐resistant breast cancer. As KDM5C's function in suppressing type I IFNs and ISGs in breast cancer is apparently dependent on its enzymatic activity, small molecule inhibitors targeting KDM5C enzymatic activity will certainly enhance tumor immunogenicity. Meanwhile, the interaction between KDM5C and ZYMND8 is also important for its function in activating estrogen/ER*α*‐target genes in breast cancer, and therefore small molecule inhibitor capable of interrupting KDM5C and ZYMND8 interaction will be efficacious in antagonizing the enhancer activation program. We propose that combination therapy with inhibitor targeting KDM5C enzymatic activity and KDM5C/ZMYND8 interaction or ER*α* antagonist would be efficacious in battling and/or provide an additional therapeutic adjunct for ER‐positive and endocrine therapy‐resistance breast cancers.

## 4 Experimental Section

##### Cloning Procedures

pBlueBac‐Flag‐KDM5C, both WT and catalytic mutant (H514A), and pFAST‐His‐ZMYND8 expression vectors were kindly provided by Dr. Yang Shi and Dr. Fei Lan, respectively, which were used as template for sub‐cloning into pCDH‐3 × Flag‐3 × HA‐EF1‐puro (System Biosciences) between BamHI and XhoI sites and pLV‐EF1‐puro(cs2.0)‐N‐3 × HA (kindly provided by Dr. Yao‐ji Liang) between BamHI and SmaI sites. KDM5C truncations, T1, T2, T3, T4, and T5, were sub‐cloned into pLV‐EF1‐puro(cs2.0)‐N‐3 × HA between BamHI and SmaI sites.

To generate siRNA‐resistant KDM5C expression constructs, both WT (KDM5C (WT)‐R) and catalytic mutant (KDM5C (H514A)‐R), siRNA targeting sequence from 5′‐CCGAGAGGAGCTAGAGCCAAA‐3′ to 5′‐CCGAGAAGAACTCGAGCCAAA‐3′ were mutated by overlapping extended PCR and sub‐cloned into pBOBI‐CS2.0 between BamHI and XhoI sites. ShRNA targeting KDM5C was cloned into pLKO.1‐TRC vector between AgeI and EcoRI sites (targeting sequence: 5′‐GCCACACTTGAGGCCATAATC‐3′ (shKDM5C); 5′‐GCAGCAGAACATTTGGAAGAA‐3′ (shKDM5C‐2)). ER*α* (full length and its truncations), CDK9, and Cyclin T1 were cloned into p3 × Flag‐CMV‐10 between EcoRI and BamHI sites. pRK5m‐CDK9‐Flag was kindly provided by Dr. Qiang Zhou. pBOBI‐3 × Flag‐hTBK1 was kindly provided by Dr. Yao‐ji Liang.

##### siRNA and Plasmids Transfection, Lenti‐Viral Vectors Packaging and Infection

SiRNAs (targeting sequence for KDM5C: 5′‐GAGAGGAGCUAGAGCCAAA‐3′ (siKDM5C); 5′‐CACACUUGAGGCCAUAAUC‐3′ (siKDM5C‐2); targeting sequence for ZMYND8: 5′‐CAUCCUUUGGUCUGGGCCA‐3′ (siZMYND8); 5′‐GGCUCGUUCUUAGACUUCU‐3′ (siZMYND8‐2)) (RiboBio) and plasmids transfection were performed using Lipofectamine 2000 (Life Technology) and polyethyleneimine (PEI, Polysciences) according to the manufacturer's protocol. Lenti‐viral vector packaging and infection was described previously.^[^ ^50^ ^]^ Briefly, HEK293T cells were seeded in culture plates coated with poly‐D‐lysine (0.1% w/v, Sigma, P7280) and transfected with lenti‐viral vectors together with packaging vectors, psPAX2 and VSVG, at a ratio of 4:3:1 using PEI (Polysciences) for 48 h according to the manufacturer's protocol. Virus was collected, filtered, and added to MCF7 cells in the presence of 10 µg mL^−1^ polybrene (Sigma, H9268). Medium was replaced 24 h later.

##### Dual Luciferase Reporter Assay

HEK293T were seeded in 24‐well plate coated with poly‐D‐lysine (0.1% w/v, Sigma, P7280) and transfected with control siRNA or siRNA specifically targeting each individual histone demethylase, and then maintained in stripping medium for 24 h before second round of siRNA transfection together with plasmid transfection including ER*α*, 2 × ERE‐TK‐luciferase and Renilla‐luciferase for another 48 h, followed by treatment with estrogen (E_2_) (10^−7^ m) for 12 h. Cells were then washed with PBS twice and lysed with 1 × passive lysis buffer(Promega) before measurement of luciferase reporter activities by using GloMax Discover Microplate Reader (Promega).

##### RNA Isolation and RT‐qPCR

Total RNA used for RT‐qPCR was purified by RNAiso Plus (TAKARA) following the manufacturer's protocol. First‐strand cDNA synthesis from total RNA was carried out using GoScript Reverse Transcription System (Promega) with DNase I treatment, followed by quantitative PCR (qPCR) using AriaMx Real‐Time PCR machine (Agilent Technologies). RNA samples from three biological repeats were pooled together and at least three technical repeats were done for each sample. Standard error of the mean is depicted. Sequence information for all primers used to check gene expression is presented in Table S2, Supporting Information. The Bliss model was used to calculate the synergistic effect (*CI*), and *CI* score less than 1 was considered to be synergistic. The synergistic effect or *CI* of double KD of siKDM5C and siZMYND8 was calculated as following: *CI* = ((siKDM5C (E_2_) + siZMYND8 (E_2_) − siKDM5C (E_2_) × siZMYND8 (E_2_)) / siKDM5C and siZMYND8 (E_2_).

##### Immunoprecipitation and Immunoblotting

Cells were lysed in lysis buffer (50 mm Tris‐HCl (pH 7.9), 150 mm KCl, 5 mm MgCl_2_, 0.2 mm EDTA, 20% glycerol, 0.1% NP‐40, and 3 mm *β*‐mercaptoethanol (*β*‐ME), complete protease inhibitor cocktail (Roche)) on ice for 30 min. Sonication (1 s on and 1 s off) was performed 20 times in 21% power (Sonics) on ice before centrifugation at 12 000 rpm for 10 min at 4 °C. For immunoprecipitation, the resultant supernatant was incubated with anti‐Flag‐M2 affinity gel (Sigma) or ‐HA agarose (Roche) in lysis buffer at 4 °C overnight. After washing 5 times with lysis buffer, resins were boiled in SDS sample buffer (1% SDS, 5% glycerol, 50 mm dithiothreitol (DTT), 30 mm Tris‐HCl, pH 6.8, and 0.25% bromophenol blue) for 5 min, resolved by 10% SDS‐PAGE gel in SDS running buffer (25 mm Tris, 250 mm glycine, and 0.1% SDS) and transferred to nitrocellulose membrane (Bio‐Rad). Immunoblotting was performed as previously described.^[^ ^50^ ^]^

For endogenous KDM5C or TBK1 immunoprecipitation, cells were lysed in lysis buffer (50 mm Tris‐HCl (pH 7.5), 250 mm NaCl, 5 mm EDTA, 0.5% NP‐40 and complete protease inhibitor cocktail (Roche)). 200 µg supernatant was subjected to immunoprecipitation with 2 µg antibodies at 4 °C overnight. Protein A/G magnetic beads (MCE) were then added and incubated at 4 °C for another 2 h, followed by washing with lysis buffer for 5 times before boiled in SDS sample buffer (1% SDS, 5% glycerol, 50 mm DTT, 30 mm Tris‐HCl, pH 6.8, and 0.25% bromophenol blue) for 5 min.

##### Cell Proliferation Assay, Fluorescence‐Activated Cell Sorting Analysis, Colony Formation Assay, and Tumor Xenograft Assay

For cell proliferation assay, MCF7 cells were transfected with siRNAs, and maintained in stripping medium for three days before treating with or without estrogen (E_2_, 10^−7^ m) for different time points followed by cell viability measurement. MCF‐7 cells infected with shRNAs were selected with 1 *μ*g mL^−1^ puromycin for two days before sub‐cultured into 96‐well plate at same density and maintained in normal growth medium for different time points followed by cell viability measurement. For control (WT) and KDM5C KD MCF7 cells, cells were seeded at the same density and maintained in normal growth medium for different time points followed by cell viability measurement. Cell viability was measured by using a CellTiter 96 AQueous one solution cell proliferation assay kit (Promega) following the manufacturer's protocol. Data were recorded at wavelength 490 nm using a GloMax Discover Microplate Reader (Promega).

For FACS analysis, cells were trypsinized, washed with PBS and fixed with ethanol at −20 °C overnight. Cells were then washed with PBS and stained with PI/Triton X‐100 staining solution (0.1% v/v Triton X‐100, 0.2 mg mL^−1^ DNase‐free RNase A (Sigma), and 0.02 mg mL^−1^ propidium iodide (Roche)) at 37 °C for 15 min. DNA content was then measured and about 10^5^ events were analyzed for each sample. Data were analyzed using ModFit LT (Verity Software House).

For colony formation assay, control (WT) and KDM5C KD MCF7 cells were seeded at the density of 1000 cells per well in a 6‐well plate, and colonies were examined 10 days after. Briefly, colonies were fixed with methanol/acid solution (3:1) for 5 min and stained with 0.1% crystal violet for 15 min.

For tumor xenograft assay, four groups (5 mice per group) of female BALB/C nude mice (age 4–6 weeks) were subcutaneously implanted with 5 × 10^6^ of control (WT) or KDM5C KD MCF7 cells suspended in PBS. Each nude mouse was brushed with estrogen (E_2_, 10^−2^ m) on the neck every 3 days for the duration of the experiments to induce tumor formation. All mice were euthanized 4 weeks after subcutaneous injection. For MMTV‐PyVT tumor xenograft assay, mammary fat pads of two groups (7 mice per group) of female FVB mice (age 5–8 weeks) were inoculated with 2 × 10^6^ of control shRNA (shCTL) and Kdm5c shRNA (shKdm5c)‐infected MMTV‐PyVT cells suspended in PBS/Matrigel GFR phenol free (Corning) in a 1:1 ratio. All mice were euthanized 23 days after inoculation. For xenograft assays to assess the effects of combination treatment, four groups (4 mice per group) of female BALB/C nude mice (age 4–6 weeks) were subcutaneously implanted with 5 × 10^6^ of MCF7 cells suspended in PBS. Estrogen was brushed on the neck every 3 days for the duration of the experiments. After 1 week, mice were randomized to treatment group based on tumor size and administrated with fulvestrant (5 mg per dose, weekly), C48 (100 mg kg^−1^, twice a day (BID)), or combination of fulvestrant and C48 for another 3 weeks. All mice were euthanized 4 weeks after subcutaneous injection. Tumors were then excised, photographed, and weighted. Animals were housed in the Animal Facility at Xiamen University under pathogen‐free conditions, following the protocol approved by the Xiamen Animal Care and Use Committee.

##### Immunohistochemistry

Tumor samples were resected and fixed in 10% formalin for 72 h. Tumor samples were then processed in ethanol and xylene and embedded in paraffin in a Leica EG1160. Paraffin blocks were sectioned at 4 microns with a Leica RM2235 rotary microtome. After antigen retrieval using EDTA antigen retrieval solution (Maxim Biotechnologies, MSV‐0098), tumor sections were stained with UltraSensitiveTM SP (Mouse/Rabbit) IHC Kit (Maxim Biotechnologies, Kit‐9730) according to the manufacturer's protocol. Primary antibodies against mouse Cd8a (eBioscience, 14‐0808‐82) were incubated at 4 °C overnight. Stained tumor sections were developed using DAB kit (Maxim Biotechnologies, DAB‐1031) for 1 min and counterstained with hematoxylin solution (Sigma, HHS16, USA) for 10 min. The IHC images analysis was performed using Cellsens Standard software (Olympus, Japan).

##### Immunofluorescence

HEK293 cells were fixed with 4% paraformaldehyde in PBS for 20 min, and then permeabilized with 0.1% Triton X‐100 in PBS on ice for 10 min. After rinsing with PBS buffer for three times, blocking solution (1% BSA in PBS) was applied for 1 h and primary antibody against Flag (Sigma, F1804, 1:200) was added in blocking buffer at 4 °C overnight. After washing with PBS/0.1% Triton X‐100 for five times, cells were incubated with DAPI and secondary antibodies conjugated with Cy3 fluorescent dyes (Beyotime, a0521, 1:500) for 1 h, washed with PBS/0.1% Triton X‐100, and mounted in Fluoromount‐G (Southern Biotech). Images were recorded on a ZEISS Exciter 5 microscope (ZEISS).

##### RNA Sequencing (RNA‐seq) and Computational Analysis of RNA‐seq Data

For RNA‐seq, total RNA was isolated using RNeasy Mini Kit (Qiagen) following the manufacturer's protocol. DNase I in column digestion was included to ensure the RNA quality. RNA library preparation was performed by using NEBNext Ultra Directional RNA Library Prep Kit for Illumina (E7420L). Paired‐end sequencing was performed at Amogene Biotech Co., Ltd. Three biological repeats were performed and then pooled together. Two such pooled sample were subjected to library construction and sequencing. Sequencing reads were aligned to hg19 reference genome by using Tophat^[^ ^51^ ^]^ (http://ccb.jhu.edu/software/tophat/index.shtml). Cuff‐diff was used to quantify the expression of RefSeq annotated genes with the option ‐M (reads aligned to repetitive regions were masked) and ‐u (multiple aligned read were corrected using “rescue method”).^[^ ^51^ ^]^ Coding genes with FPKM (fragments per kilobase per million mapped reads) larger than 0.5, either in control or estrogen‐treated sample, were included in the analysis. Estrogen‐regulated gene program was determined by fold change (*FC*) of FPKM for genes in control and estrogen‐treated samples (*FC* > 1.5). FPKM of a gene was calculated as mapped reads on exons divided by exonic length and the total number of mapped reads. Box plots were generated by R software and significance was determined using Student's *t*‐test. Heat maps were visualized using Java TreeView.

All RNA‐seq data were deposited in the Gene Expression Omnibus database under accession GSE141988.

##### Chromatin Immunoprecipitation Coupled with High Throughput Sequencing (ChIP‐seq), ChIP‐qPCR, and Computational Analysis of ChIP‐seq Data

Cells were maintained in stripping medium for three days and then treated with or without estrogen (E_2_, 10^−7^ m) for 1 h before ChIP or ChIP‐seq. Cells were fixed with 1% formaldehyde (Sigma) for 10 min at room temperature (RT) (H3K4me3 (Abcam, ab8580 and H3K4me2 (Abcam, ab7766) ChIP), or with disuccinimidyl glutarate (2 mm) (Proteochem) for 45 min at RT, washed twice with PBS and then double‐fixed with 1% formaldehyde for another 10 min at RT (KDM5C (Bethyl Laboratory, A301‐034A) and ZMYND8 (Bethyl Laboratory, A302‐090A) ChIP), or fixed with 1% glutaraldehyde for 10 min at RT (CDK9 (CST, 2316S) ChIP). Fixation was stopped by adding glycine (0.125 m) and incubated for 5 min at RT, followed by washing with cold PBS twice. Chromatin DNA was sheared to 200–500 bp average in size through sonication in ChIP lysis buffer (1% SDS, 10 mm EDTA, 50 mm Tris‐HCl pH 7.8, and 1 × complete protease inhibitor cocktail (Roche)) as described previously.^[^ ^50^, ^52^ ^]^ Briefly, resultant was diluted in dilution buffer (1% Triton X‐100, 2 mm EDTA, 150 mm NaCl, 20 mm Tris‐HCl pH 7.8, and 1 × complete protease inhibitor cocktail) and immunoprecipitated with specific antibodies overnight at 4 °C, followed by incubation with protein G magnetic beads (Bio‐Rad, 161‐4023) for an additional 3 h. The bound fractions were sequentially washed for 15 min at 4 °C with TSEI (0.1% SDS, 1% Triton X‐100, 2 mm EDTA, 150 mm NaCl, 20 mm Tris‐HCl pH 7.8, and 1 × complete protease inhibitor cocktail), TSEII (0.1% SDS, 1% Triton X‐100, 2 mm EDTA, 400 mm NaCl, 20 mm Tris‐HCl pH 7.8, and 1 × complete protease inhibitor cocktail), and TE buffer (20 mm Tris‐HCl pH 7.4, and 1 mm EDTA). Formaldehyde or double crosslinked chromatin was treated at 65 °C overnight whereas glutaraldehyde crosslinked chromatin was treated with 0.2 mg mL^−1^ proteinase K at 55 °C for 2 h, then 65 °C overnight with interval shaking. Immunoprecipitated DNA was purified by using QIAquick spin columns (Qiagen) and subjected to high throughput sequencing.

For all ChIP‐seq done in this manuscript, two biological repeats were performed and then pooled for library preparation. ChIP‐seq sample preparation and computational analysis of ChIP‐seq data were performed as following.

The libraries were constructed following NEB Ultra II DNA Library Prep Kit for Illumina (E7645s). Briefly, ChIP DNA was end‐blunted and added with an “A” base so adaptors from the NEBNext Adaptor for Illumina with a “T” could ligate on the ends. Then 200–400 bp fragments were gel‐isolated and purified. The library was amplified by 18 cycles of PCR.

The image analysis and base calling were performed by using Illumina's Genome Analysis pipeline. The sequencing reads were aligned to hg19 reference genome by using Bowtie2^[^ ^53^ ^]^ (http://bowtie-bio.sourceforge.net/bowtie2/index.shtml) with default parameters. Both uniquely and multiply aligned reads were kept for downstream analysis (if a read aligned to multiple genomic locations, only one location with the best score was chosen). Clonal amplification was circumvented by allowing maximal one tag for each unique genomic position. The identification of ChIP‐seq peaks was performed using HOMER.^[^ ^54^ ^]^ The threshold for the number of tags that determined a valid peak was selected at a false discovery rate (*FDR*) of 0.001. Fourfold more tags relative to the local background region (10 kb) were also required to avoid identifying regions with genomic duplications or non‐localized binding. Genomic distribution was done by using the default parameters from HOMER with minor modifications, in which promoter peaks were defined as those with peak center falling between 1000 bp downstream and 5000 bp upstream of transcript start sites. To define estrogen‐induced KDM5C binding sites, only those KDM5C binding sites that were co‐localized with ER*α* under estrogen treatment were considered as the candidates. Motif analysis was performed using HOMER. Tag density for histograms (50 bp per bin), box plots, and heat maps were generated by using HOMER. Box plots were then generated by R software (https://www.r-project.org/) and significance was determined using Student's *t*‐test. Heat maps were visualized using Java TreeView^[^ ^55^ ^]^ (http://jtreeview.sourceforge.net). Metagenes of ChIP‐seq tag density across genes were generated by using deepTools.

ER*α* ChIP‐seq was from GSE45822; H3K27Ac and P300 ChIP‐seq were from GSE62229; H3K4me3, H3K9me3, and H3K27me3 ChIP‐seq were from GSE23701. ChIP‐seq was deposited in the Gene Expression Omnibus database under accession GSE141988.

##### Generation of KDM5C Knockdown Cell Lines Using CRISPR/Cas9 Gene Editing Technology

KDM5C KD MCF7 cells were generated by using CRISPR/Cas9 system. Specifically, gRNA sequence (5′‐GGTAGGAAATCGTCGGACCC‐3′ (gRNA); 5′‐CGGTGGCGGTAGGAAATCGT‐3′ (gRNA‐2)) targeting KDM5C was first cloned into pX330‐U6‐Chimeric_BB‐CBh‐hSpCas9 (Addgene, 42230) (pX330‐gRNA‐KDM5C), which was then transfected into MCF7 cells together with pIREShyg3 vector expression hygromycin (Clontech, 631620), followed by hygromycin B (0.2 mg mL^−1^) selection until formation of single colonies. Single colonies were then subjected to immunoblotting to select the ones with KDM5C KD.

##### Stable Isotope Labeling by Amino Acids in Cell Culture, Affinity Purification, In Solution Digestion, and LC‐MS/MS Analysis

Cell labeling was done by SILAC (stable isotope labeling by amino acids in cell culture). MCF7 cells were grown in SILAC DMEM (Invitrogen) supplemented with L‐lysine/arginine‐U‐^13^C_6_ (heavy label) (Cambridge Isotope Laboratories) and 10% dialyzed fetal bovine serum, L‐glutamine, and penicillin/streptomycin for 2 weeks, then infected with lenti‐viral vectors expressing pCDH‐CMV‐3 × Flag‐KDM5C‐3 × HA‐EF1‐Puro for 48 h before adding estrogen (10^−7^ m, 1 h). Viruses were packaged in HEK293T cells labeled similarly as MCF7 cells. Serving as a control, MCF7 cells grown in normal medium containing L‐lysine/arginine (light label) (Sigma) were infected with viruses packaged in HEK293T cells grown in normal medium and treated with ethanol. Ethanol and estrogen‐treated MCF7 cells were pooled and subjected to nuclear extract preparation by using buffer A containing 10 mm HEPES‐KOH pH 7.8, 1.5 mm MgCl_2_, 10 mm KCl, 1 mm DTT, and 1 × complete protease inhibitor cocktail. Nuclear extracts were lysed in buffer C containing 20 mm HEPES‐KOH pH 7.8, 25% glycerol, 420 mm NaCl, 1.5 mm MgCl_2_, 0.2 mm EDTA pH 8.0, 1 mm DTT, and 1 × complete protease inhibitor cocktail and subjected to affinity purification by using anti‐HA‐agarose, washed extensively three times with a buffer D (0.15) containing 0.15 m KCl, 20 mm HEPES‐KOH pH 7.8, 15% glycerol, 0.2 mm EDTA pH 8.0, 0.2% NP‐40, 1 mm DTT, and 1 × complete protease inhibitor cocktail, and two times with a buffer D (0.1) containing 0.1 m KCl, 20 mm HEPES‐KOH pH 7.8, 15% glycerol, 0.2 mm EDTA pH 8.0, 0.2% NP‐40, 1 mm DTT, and 1 × complete protease inhibitor cocktail and eluted with HA peptides (Sigma). Elutes were then subjected to in solution digestion and LC‐MS/MS analysis following the protocol described previously.^[^ ^50^ ^]^ Briefly, the elutes after IP were first reduced in 20 mm DTT (Sigma) at 95 °C for 5 min, and subsequently alkylated in 50 mm iodoacetamide (Sigma) for 30 min in the dark at RT. After alkylation, the samples were transferred to a 10 kD centrifugal spin filter (Millipore) and sequentially washed with 200 µL of 8 m urea for three times and 200 µL of 50 mm ammonium bicarbonate for two times by centrifugation at 14 000 g. Next, tryptic digestion was performed by adding trypsin (Promega) at 1:50 (enzyme/substrate, m/m) in 200 µL of 50 mm ammonium bicarbonate at 37 °C for 16 h. Peptides were recovered by transferring the filter to a new collection tube and spinning at 14 000 g. To increase the yield of peptides, the filter was washed twice with 100 µL of 50 mm ammonium bicarbonate. Peptides were desalted by StageTips. MS experiments were performed on a nanoscale UHPLC system (EASY‐nLC1000, Proxeon Biosystems) connected to an Orbitrap Q‐Exactive equipped with a nanoelectrospray source (Thermo Fisher Scientific). The peptides were dissolved in 0.1% formic acid (FA) with 2% acetonitrile (ACN) and separated on a RP‐HPLC analytical column (75 µm × 15 cm) packed with 2 µm C18 beads (Thermo Fisher Scientific) using a 4 h gradient ranging from 5% to 35% ACN in 0.1% FA at a flow rate of 300 nL min^−1^. The spray voltage was set at 2.5 kV and the temperature of ion transfer capillary was 275 °C. A full MS/MS cycle consisted of one full MS scan (resolution, 70 000; automatic gain control (AGC) value, 1e6; maximum injection time, 100 ms) in profile mode over a mass range between *m*/*z* 350 and 1800, followed by fragmentation of the top twenty most intense ions by high energy collisional dissociation with normalized collision energy at 28% in centroid mode (resolution, 17 500; AGC value: 1e5, maximum injection time: 100 ms). The dynamic exclusion window was set at 30 s. One microscan was acquired for each MS and MS/MS scan. Unassigned ions or those with a charge of 1+ and > 7+ were rejected for MS/MS. Raw data was processed using Proteome Discoverer (PD, version 2.1), and MS/MS spectra were searched against the reviewed Swiss‐Prot human proteome database. All searches were carried out with precursor mass tolerance of 10 ppm, fragment mass tolerance of 0.02 Da, oxidation (Met) (+15.9949 Da), methylation (Arg, Lys) (+14.0266 Da), dimethylation (Arg, Lys) (+28.0532 Da), and acetylation (protein N‐terminus) (+42.0106 Da) as variable modifications, carbamidomethylation (Cys) (+57.0215 Da) as fixed modification and three trypsin missed cleavages allowed. Only peptides with at least six amino acids in length were considered. The peptide and protein identifications were filtered by PD to control the *FDR* < 1%. At least one unique peptide was required for protein identification.

##### Protein Purification, In Vitro Pull‐Down Assay, In Vitro Demethylation Assay, and In Vitro Phosphorylation Assay

pBlueBac‐Flag‐KDM5C was expressed in Sf9 cells and purified as following. Briefly, cells expressing Flag‐tagged KDM5C were lysed in lysis buffer containing 20 mm Tris‐HCl pH 7.5, 150 mm NaCl, 0.1% NP‐50, 0.2% Triton X‐100, 1 mm DTT, and EDTA‐free protease inhibitor cocktail, and purified as described previously.^[^ ^11^ ^]^ HA‐tagged KDM5C and ZMYND8, and Flag‐tagged ER*α*, CDK9, Cyclin T1 and TBK1 were expressed in HEK293T and lysed in a lysis buffer containing 50 mm Tris HCl (pH 7.4), 420 mm NaCl, 1 mm EDTA, and 1% Triton X‐100 supplemented with complete protease inhibitor cocktail for in vitro pull‐down assay, and EDTA‐free protease inhibitor cocktail for enzymatic assay.

For in vitro pull‐down assay, HA‐tagged KDM5C or ZMYND8 immobilized on anti‐HA agarose (clone HA‐7) were incubated with Flag‐tagged CDK9, Cyclin T1, TBK1, or KDM5C eluted with 3 × Flag peptide (Sigma) and concentrated with Amicon Ultra‐0.5 mL centrifugal filters(Millipore) in binding buffer containing 50 mm Tris‐HCl pH 7.9, 150 mm KCl, 5 mm MgCl_2_, 0.2 mm EDTA, 20% glycerol, 0.1% NP‐40, 3 mm *β*‐ME, and complete protease inhibitors cocktail at 4 °C for 4 h. Anti‐HA agarose was then washed 5 times with binding buffer at 4 °C and boiled for 5 min in 1 × SDS sample buffer for SDS‐PAGE gel and immunoblotting analysis.

For in vitro demethylation assay, Flag‐tagged KDM5C was incubated with or without Flag‐tagged ER*α* in demethylation reaction buffer (20 mm Tris‐HCl pH 7.5, 150 mm NaCl, 50 *μ* m [NH_4_]_2_Fe[SO_4_]_2_, 1 mm *α*‐ketoglutarate, and 2 mm ascorbic acid) at RT for 15 min before adding 1 *μ*g of synthetic peptide with methylated H3K4me3 at 37 °C for another 1 h. The reactions were then subjected to MALDI‐TOF MS analysis as described previously.

For in vitro phosphorylation assay, Flag‐tagged TBK1 was incubated with or without Flag‐tagged KDM5C or GST control proteins in phosphorylation reaction buffer (50 mm HEPES pH 7.5, 5 mm MgCl_2_) with or without 30 mm ATP at 30 °C for 30 min. The reactions were terminated by adding 1 × SDS sample buffer for SDS‐PAGE gel and immunoblotting analysis.

##### Statistical Analysis

Gene expression data were normalized to ACTIN, and ChIP‐qPCR data were normalized to input DNA. Data are presented as mean ± SD, and sample size for each statistical analysis is indicated in the appropriate figure legends. Comparison of two groups or data points was performed by using two‐tailed Student's *t*‐test. *p*‐value < 0.05 were considered statistically significant. Survival curves were constructed according to the Kaplan–Meier method and compared using log‐rank (Mantel‐Cox) test.

## Conflict of Interest

The authors declare no conflict of interest.

## Author Contributions

W.‐j.Z., Y.H., Y.‐h.H., G.‐s.H., contributed equally to this work. W.L. conceived the original ideas, designed the project, and wrote the manuscript with inputs from M.G.R., H.‐f.S., W.‐j.Z., Y.H., Y.‐h.H., G.‐s.H., L.W., B.‐l.P., J.Y., W.‐j.L., and S.‐W.L. H.S. performed the majority of the experiments with participation from Y.‐h.H., G.‐s.H., Y.H., L.W., B.‐l.P., J.Y., T.‐t.L., R.R., X.‐y.C., J.‐y.L., W.‐j.L., and K.O. W.‐j.Z. and G.‐s.H. performed all the bioinformatics analyses.

## Supporting information

Supporting Information

Supporting Information

Supporting Information

Supporting Information

## Data Availability

All RNA‐seq data were deposited in the Gene Expression Omnibus database under accession GSE141988. The following link has been created to allow review of record GSE141988 while it remains in private status: https://www.ncbi.nlm.nih.gov/geo/query/acc.cgi?acc=GSE141988 (token: ktohieuglpmpvgl) ERï i ChIP‐seq was from GSE45822; H3K27Ac and P300 ChIP‐seq were from GSE62229; H3K4me3, H3K9me3 and H3K27me3 ChIP‐seq were from GSE23701. ChIP‐seq was deposited in the Gene Expression Omnibus database under accession GSE141988. The following link has been created to allow review of record GSE141988 while it remains in private status: https://www.ncbi.nlm.nih.gov/geo/query/acc.cgi?acc=GSE141988 (token: ktohieuglpmpvgl)
