# Supplementary material for: The Dual Function of KDM5C in Both Gene Transcriptional Activation and Repression Promotes Breast Cancer Cell Growth and Tumorigenesis
Source: Adv Sci (Weinh). 2021 Feb 18;8(9):2004635. doi: 10.1002/advs.202004635 (PMC8097366; doi:10.1002/advs.202004635)
Supplement: Supplementary file 1 — Supporting Information [file ADVS-8-2004635-s004.pdf]

© 2021 Wiley-VCH GmbH

ADVANCED SCIENCE
Open Access

Supporting Information

for *Adv. Sci.*, DOI: 10.1002/advs.202004635

The Dual Function of KDM5C in Both Gene Transcriptional Activation and Repression Promotes Breast Cancer Cell Growth and Tumorigenesis

Hai-feng Shen, Wen-juan Zhang, Ying Huang, Yao-hui He, Guo-sheng Hu, Lei Wang, Bing-ling Peng, Jia Yi, Ting-ting Li, Rui Rong, Xiao-yan Chen, Jun-yi Liu, Wen-juan Li, Kenny Ohgi, Shao-Wei Li, Michael G. Rosenfeld, and Wen Liu*

Supplementary Information

Supplementary Figure Legends

Figure S1. KDM5C is required for ERα-positive breast cancer cell proliferation.

(A) MCF7 cells were transfected with control siRNA (siCTL) or siRNA specifically against each individual member in the histone demethylase protein family, both LSD (LSD1 and LSD2) and JmjC domain-containing protein subfamilies, and then treated with or without estrogen (E_2) as indicated followed by cell proliferation assay. Those demethylases required for estrogen-induced MCF7 cell growth were indicated by brackets (± SD., *P < 0.05, **P < 0.01, ***P < 0.001).

(B) Kaplan Meier survival analyses for OS (overall survival) of ER-negative breast cancer patients using KDM5C as input (n=173).

(C, D) T47D (C) and BT474 (D) cells were transfected with siCTL or siKDM5C in stripping medium for three days, and then treated with or without estrogen (E_2, 10^{-7} M) for duration as indicated followed by cell proliferation assay (± SD., *P < 0.05, **P < 0.01, ***P < 0.001).

(E) MCF7 cells were transfected with control siRNA (siCTL) or a second independent siRNA specific against *KDM5C* (siKDM5C-2) in stripping medium for three days, and then treated with or without estrogen (E_2, 10^{-7} M) for different duration as indicated followed by cell proliferation assay (± SD, **P < 0.01, ***P < 0.001).

(F) MCF7 cells were transfected with siCTL or a second independent siRNA specific against *KDM5C* (siKDM5C-2) in stripping medium for three days, and then treated with or without estrogen (E_2, 10^{-7} M) for 24 hrs followed by FACS analysis.

(G) MCF7 cells transfected with siCTL or siKDM5C-2 were subjected to immunoblotting (IB) using antibodies as indicated.

(H) MCF7 cells were infected with control shRNA (shCTL) or a second independent shRNA specific against KDM5C (shKDM5C-2) lenti-virus for duration as indicated followed by cell proliferation assay (± SD, $**P < 0.01$, $***P < 0.001$).

(I) MCF7 cells infected with shCTL or shKDM5C-2 were subjected to immunoblotting (IB) using antibodies as indicated.

(J) Wild type (WT) and KDM5C knockdown (KDM5C (sgRNA-2)) MCF7 cells generated by CRISPR/Cas9 were subjected to cell proliferation assay (± SD, $**P < 0.01$).

(K) WT and KDM5C (sgRNA-2) MCF7 cells were subjected to immunoblotting using antibodies as indicated.

Figure S2. KDM5C activates estrogen/ERα-target genes.

(A) Correlation of KDM5C's effects on estrogen response between two RNA-seq biological repeats.

(B, C) UCSC Genome browser views of RNA-seq as described in Fig. 2A for two estrogen/ERα-target genes, *PGR* (B) and *NRIP1* (C), were shown.

(E) MCF7 cells were transfected with siCTL or a second independent siKDM5C (siKDM5C-2) in stripping medium for three days, and treated with or without estrogen (E_2, 10^{-7} M, 6 hrs), followed by RNA extraction and RT-qPCR analysis to examine the expression of selected estrogen-induced genes as indicated.

(H) MCF7 cells were infected with shCTL or a second independent shKDM5C (shKDM5C-2) lenti-virus in stripping medium for three days, and treated with or without estrogen (E_2, 10^{-7} M, 6 hrs), followed by RNA extraction and RT-qPCR analysis to examine the expression of selected estrogen-induced genes as indicated.

(K) WT and KDM5C (sgRNA-2) MCF7 cells were maintained in stripping medium for three days, and treated with or without estrogen (E_2, 10^{-7} M, 6 hrs), followed by RNA extraction and RT-qPCR analysis to examine the expression of selected estrogen-induced genes as indicated.

(M, O) T47D (M) and BT474 (O) cells were transfected with siCTL or siKDM5C in stripping medium for three days, and treated with or without estrogen (E_2, 10^{-7} M, 6 hrs), followed by RNA extraction and RT-qPCR analysis to examine the expression of selected estrogen-induced genes as indicated.

(D, F, G, I, J, L, N, P) Corresponding values for heat map shown in Fig. 2F (D), Fig. S2E (F), Fig. 2G (G), Fig. S2H (I), Fig. 2H (J), Fig. S2K (L), Fig. S2M (N) and Fig. S2O (P). Significance test shown on the very right was performed for the difference between siCTL (E_2) and siKDM5C (E_2), shKDM5C (E_2) or KDM5C (sgRNA) samples (± SD, n.s: no significance, *P < 0.05, **P < 0.01, ***P < 0.001).

Figure S3. KDM5C represses type I IFNs and ISGs expression.

(A) Correlation of KDM5C's effects on gene expression between two RNA-seq biological repeats.

(E) MCF7 cells were transfected with siCTL or a second independent siKDM5C (siKDM5C-2) for three days, followed by RNA extraction and RT-qPCR analysis to examine the expression of selected type I IFNs and ISGs as indicated.

(G) MCF7 cells were infected with shCTL or a second independent shKDM5C (shKDM5C-2) lenti-virus for three days, followed by RNA extraction and RT-qPCR analysis to examine the expression of selected type I IFNs and ISGs as indicated.

(I) Wild type (WT) and KDM5C knockdown (KDM5C (sgRNA)) MCF7 cells were subjected to RNA extraction and RT-qPCR analysis to examine the expression of selected type I IFNs and ISGs as indicated.

(K, M) T47D (K) and BT474 (M) cells were transfected with siCTL or siKDM5C for three days, followed by RNA extraction and RT-qPCR analysis to examine the expression of selected type I IFNs and ISGs as indicated.

(B, C, D, F, H, J, L, N) Corresponding values for heat map shown in Fig. 3F (B), 3G (C), 3H (D), S3E (F), S3G (H), S3I (J), S3L (K) and Fig. S3N (M) (± SD, *P < 0.05, **P < 0.01, ***P < 0.001).

Figure S4. KDM5C is recruited to ERα-bound active enhancers upon estrogen stimulation.

(A) ChIP-seq tag distribution, including ERα, H3K4me1, H3K4me2, H3K4me3, H3K27Ac, P300, H3K9me3 and H3K27me3, centered on all of its own sites (left panels) or estrogen-induced KDM5C sites (right panels) (± 3,000 bp).

(B, C) UCSC Genome browser views of KDM5C, ERα, H3K4me1, H3K4me2, H3K4me3, H3K27Ac, P300, H3K9me3 and H3K27me3 ChIP-seq in the presence and absence of estrogen on selected active enhancer regions in the vicinity of estrogen-induced target genes, *SIAH2* (B) and *P2RY2* (C). Boxed regions indicated active enhancers.

Figure S5. The sequence of recruitment of ERα, ZMYND8, KDM5C and P300 onto ERα-bound active enhancers.

(A) MCF7 cells were subjected to cellular fractionation. Both nuclear and cytosolic fractions were subjected to immunoblotting (IB) analysis using antibodies as indicated. PARP1 and HSP60 were served as purity control for nuclear and cytosolic fractions, respectively.

(B) MCF7 cells transfected with siCTL or siZMYND8 were treated with estrogen (E_2, 10^{-7} M, 1 hr) followed by ChIP with anti-KDM5C specific antibody. KDM5C binding was examined on enhancer (E) regions nearby estrogen-target genes as indicated. Data presented was fold change of ChIP signals over siCTL after normalized to input (± SD, **P < 0.01, ***P < 0.001).

(D) MCF7 cells were treated with or without fulvestrant (ICI 182,780, ICI, 100 nM, 12 hrs) before adding estrogen (E_2, 10^{-7} M, 1 hr) followed by ChIP with anti-ZMYND8 specific antibody. ZMYND8 binding was examined on enhancer (E) regions nearby estrogen-target genes as indicated. Data presented was fold change of ChIP signals over siCTL after normalized to input (± SD, **P < 0.01, ***P < 0.001).

(F) MCF7 cells were subjected to immunoprecipitation (IP) with anti-P300 antibody followed by immunoblotting (IB) analysis as indicated.

(G) MCF7 cells transfected with siCTL or siZMYND8 were treated with estrogen (E_2, 10^{-7} M, 1 hr) followed by ChIP with anti-P300 specific antibody. P300 binding was examined on enhancer (E) regions nearby estrogen-target genes as indicated. Data presented was fold change of ChIP signals over siCTL after normalized to input (± SD, **P < 0.01, ***P < 0.001).

(I) MCF7 cells transfected with siCTL or siKDM5C were treated with estrogen (E_2, 10^{-7} M, 1 hr) followed by ChIP with anti-P300 specific antibody. P300 binding was examined on enhancer (E) regions nearby estrogen-target genes as indicated. Data presented was fold change of ChIP signals over siCTL after normalized to input (± SD, **P < 0.01, ***P < 0.001).

(C, E, H, J) Corresponding values for heat map shown in Fig. S5B (C), Fig. S5D (E), Fig. S5G (H) and Fig. S5I (J) (± SD, **P < 0.01, ***P < 0.001).

Figure S6. ZMYND8 is involved in KDM5C-activated estrogen gene expression.

(A, B) Heat map (A) and box plot (B) representation of the expression (FPKM, log2) for genes induced by estrogen (E_2) and dependent on both KDM5C and ZMYND8 as described in Fig. 5L.

(C, F, I, K, M) Corresponding values for heat map shown in Fig. 5M (C), Fig. S6E (F), Fig. S6H (I), Fig. S6J (K) and Fig. S6L (M) (± SD, ns indicates no significance, *P < 0.05, **P < 0.01, ***P < 0.001).

(D) MCF7 cells transfected with siCTL or siZYMND8 were subjected to immunoblotting (IB) analysis. GAPDH was served as a loading control.

(E) MCF7 cells transfected with control siRNA (siCTL) or a second independent siRNA targeting ZMYND8 (siZYMND8-2) were treated with or without estrogen (E_2, 10^{-7} M, 6 hrs) followed by RNA extraction and RT-qPCR analysis to examine the expression of selected estrogen-target genes as indicated.

(G) MCF7 cells transfected with siCTL or a second independent siRNA targeting ZMYND8 (siZYMND8-2) were subjected to immunoblotting (IB) analysis. GAPDH was served as a loading control.

(H, J) T47D (H) and BT474 (J) cells were transfected with siCTL or siZMYND8 in stripping medium for three days, and then treated with or without estrogen (E_2, 10^{-7} M, 6 hrs) followed by RNA extraction and RT-qPCR analysis to examine the expression of selected estrogen-target genes as indicated.

(L) MCF7 cells were transfected with siCTL, siKDM5C and siZMYND8 alone or in combination in stripping medium for three days, and then treated with or without estrogen (E_2, 10^{-7} M, 6 hrs) followed by RNA extraction and RT-qPCR analysis to examine the expression of selected estrogen-target genes as indicated.

Figure S7. ZMYND8 is involved in KDM5C-repressed type I IFNs and ISGs expression.

(A, B) Heat map (A) and box plot (B) representation of the expression (FPKM, log2) for genes commonly-repressed by both KDM5C and ZMYND8 as described in Fig. 5N.

(C, E, G) Corresponding values for heat map shown in Fig. 5O (C), Fig. S7D (E) and Fig. S7F (G) (± SD, $*P < 0.05$, $**P < 0.01$, $***P < 0.001$).

(D) MCF7 cells were infected with siCTL or a second independent siZMYND8 (siZMYND8-2) for three days, followed by RNA extraction and RT-qPCR analysis to examine the expression of selected type I IFNs and ISGs as indicated.

(F) T47D cells were transfected with siCTL or siZMYND8 for three days, followed by RNA extraction and RT-qPCR analysis to examine the expression of selected type I IFNs and ISGs as indicated.

Figure S8. ZMYND8 is required for the growth of ERα-positive breast cancer.

(A) MCF7 cells were transfected with siCTL or a second independent siZYMND8 (siZYMND8-2) in stripping medium for three days, and then treated with or without estrogen (E_2, 10^{-7} M) for different duration as indicated followed by cell proliferation assay (± SD, ***P < 0.001).

(B, C) T47D (B) and BT474 (C) cells were transfected with siCTL or siZYMND8 in stripping medium for three days, and then treated with or without estrogen (E_2, 10^{-7} M) for different duration as indicated followed by cell proliferation assay (± SD, *P < 0.05, **P < 0.01, ***P < 0.001).

Figure S9. KDM5C activates ERα-target genes in an enzymatic-independent manner.

(A) Corresponding values (± SD) for heat map shown in Fig. 6A.

(B) MCF7 cells were transfected with control siRNA (siCTL) or siRNA specific against KDM5C (siKDM5C) in stripping medium for three days, and treated with or without estrogen (E_2, 10^{-7} M, 1 hr) followed by H3K4me3 ChIP analysis. The occupancy of H3K4me3 was examined for selected active enhancer regions in the vicinity of estrogen-induced genes as indicated.

(C) Corresponding values (± SD) for heat map shown in (B).

(D) ChIP samples as described in (B) was used to examine the occupancy of H3K4me3 on *TFF1* promoter region (± SD, **P < 0.01, ***P < 0.001).

(E) Cells as described in (B) were subjected to immunoblotting (IB) analysis to examine the levels of H3K4me3 and histone H3.

(F) Corresponding values (± SD) for heat map shown in Fig. 6B.

(G) MCF7 cells were transfected with siCTL or siKDM5C in the presence or absence of control vector, KDM5C (WT)-R or KDM5C (H514A)-R in stripping medium for three days, and then treated with or without estrogen (E_2, 10^{-7} M, 6 hrs) followed by RNA extraction and RT-qPCR analysis to examine the expression of selected estrogen-target genes as indicated. R, siRNA-resistant.

(H) Corresponding values (± SD) for heat map shown in (G) (± SD, ns, non-significant, *P < 0.05, **P < 0.01, ***P < 0.001).

(I) *In vitro* demethylation assay was performed by mixing H3K4me3 peptide (mass to charge (M/Z): 2260) with *in vitro* purified KDM5C in the presence or absence of ERα followed by MALDI-TOF MS analysis. H3K4me3 peptide (M/Z: 2260) as well as its demethylated products, H3K4me2 (M/Z: 2246) and H3K4me1 (M/Z: 2232), were shown.

Figure S10. KDM5C interacts with TBK1 to inhibit its phosphorylation and the expression of type I IFNs and ISGs.

(A) MCF7 cells were infected with lenti-viral vectors expressing Flag-tagged KDM5C followed by immunofluorescence analysis. Red, Flag-tagged proteins; Blue, DAPI.

(B) *In vitro* phosphorylation assay was performed by mixing *in vitro* purified Flag-tagged TBK1 and GST proteins, followed by immunoblotting (IB) analysis with antibodies as indicated. p-TBK1: anti-TBK1 phosphorylated at serine 172 antibody.

(C) Corresponding values (± SD) for heat map shown in Fig. 8K.

Figure S11. Simultaneously blocking estrogen/ERα-target gene expression by ERα antagonist and Type I IFNs and ISGs expression by KDM5 inhibitor is effective in suppressing ERα-positive breast cancer cell growth.

(A, B) T47D (A) or BT474 (B) cells cultured in the presence of estrogen (E_2, 10^{-7} M) were treated with or without fulvestrant (ICI, 0.1 μM for T47D, 1 μM for BT474), C70 (5 μM) or C48 (10 μM) alone or in combination for duration as indicated followed by cell proliferation assay.

Figure S12. A proposed model of KDM5C function in gene transcriptional regulation in ERα-positive breast cancer cells.

KDM5C activates estrogen/ERα-target genes and represses type I IFNs and ISGs via distinct mechanisms, with KDM5C binds to ERα-bound active enhancers and activates estrogen/ERα-target genes directly in an enzymatic-independent manner, while represses type I IFNs and ISGs indirectly in an enzymatic-dependent manner through inhibiting TBK1 phosphorylation. KDM5C's dual activities in gene transcriptional regulation together promote breast cancer cell growth and tumorigenesis.

Supplementary Table Legend

Table S1. Proteins associated with KDM5C in both cytosol and nucleus identified by mass spectrometry.

Table S2. Sequence information for all qPCR primers used in the current study. Sequence information of qPCR primers designed to detect gene expression (mRNA) or factor binding on chromatin (ChIP) were shown. F: forward; R: reverse.

Table S3. Antibodies used in the current study. Vendor, catalog number and applications of antibodies used in this study were shown. IB: immunoblotting; IP: immunoprecipitation; ChIP-

seq: chromatin immunoprecipitation coupled with high throughput sequencing; IHC: Immunohistochemistry.

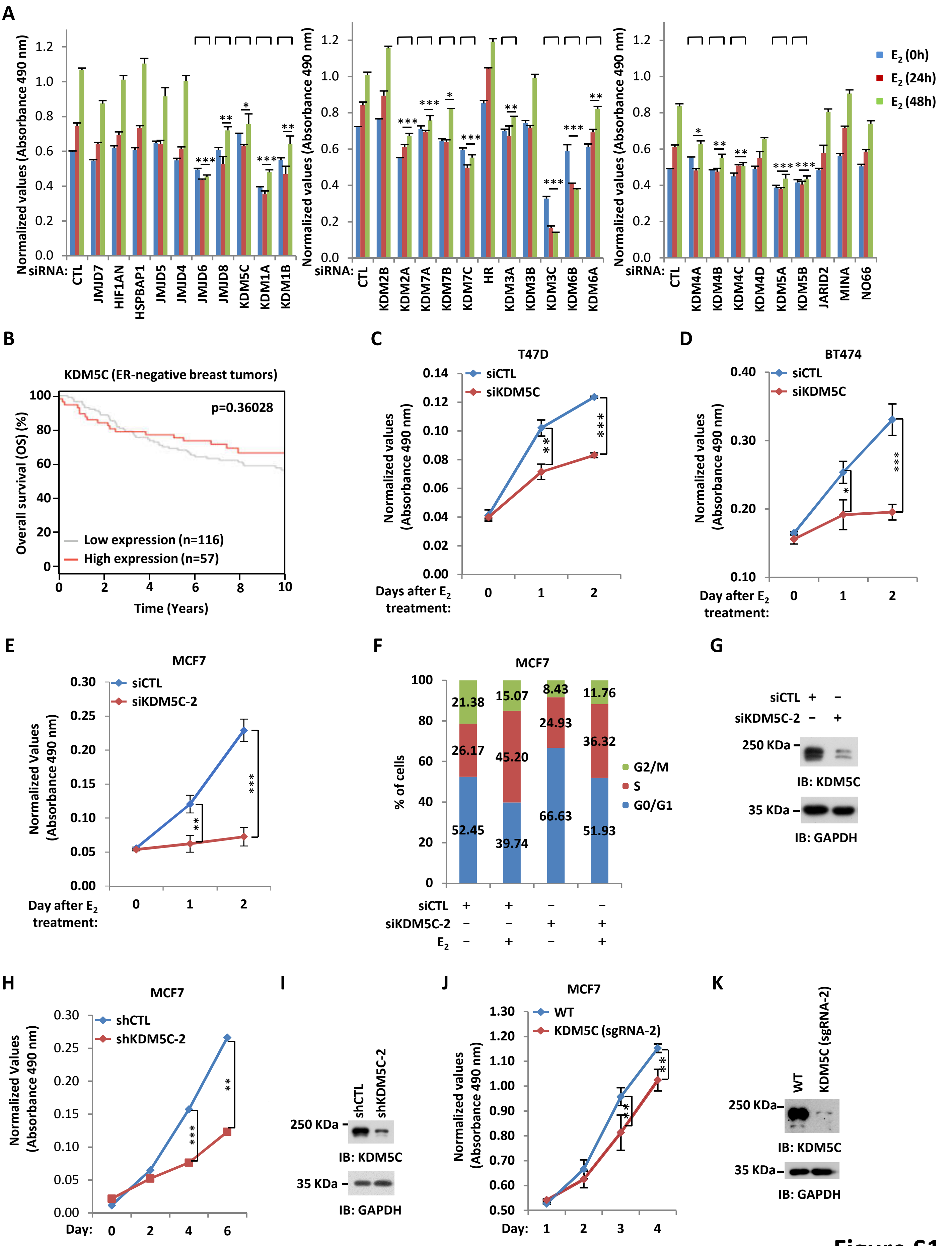

A
Normalized values (Absorbance 490 nm)
siRNA: CTL JMJD7 HIF1AN HSPBAP1 JMJD5 JMJD4 JMJD6 JMJD8 KDM5C KDM1A KDM1B
siRNA: CTL KDM2B KDM2A KDM7A KDM7B KDM7C HR KDM3A KDM3B KDM3C KDM6B KDM6A
siRNA: CTL KDM4A KDM4B KDM4C KDM4D KDM5A KDM5B JARID2 MINA NO66
E_2 (0h)
E_2 (24h)
E_2 (48h)
B
KDM5C (ER-negative breast tumors)
p=0.36028
Overall survival (OS) (%)
Low expression (n=116)
High expression (n=57)
Time (Years)
C
T47D
siCTL
siKDM5C
Normalized values (Absorbance 490 nm)
Days after E_2 treatment:
D
BT474
siCTL
siKDM5C
Normalized values (Absorbance 490 nm)
Day after E_2 treatment:
E
MCF7
siCTL
siKDM5C-2
Normalized Values (Absorbance 490 nm)
Day after E_2 treatment:
F
MCF7
% of cells
21.38 15.07 8.43 11.76
26.17 45.20 24.93 36.32
52.45 39.74 66.63 51.93
G2/M
S
G0/G1
siCTL + + – –
siKDM5C-2 – – + +
E_2 – + – +
G
siCTL + –
siKDM5C-2 – +
250 KDa
IB: KDM5C
35 KDa
IB: GAPDH
H
MCF7
shCTL
shKDM5C-2
Normalized Values (Absorbance 490 nm)
Day:
I
shCTL
shKDM5C-2
250 KDa
IB: KDM5C
35 KDa
IB: GAPDH
J
MCF7
WT
KDM5C (sgRNA-2)
Normalized values (Absorbance 490 nm)
Day:
K
WT
KDM5C (sgRNA-2)
250 KDa
IB: KDM5C
35 KDa
IB: GAPDH

Figure S1

A

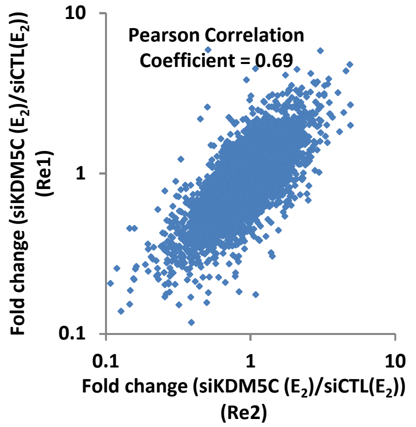

B

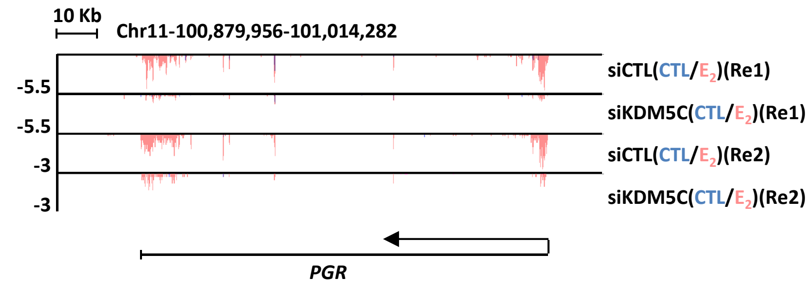

C

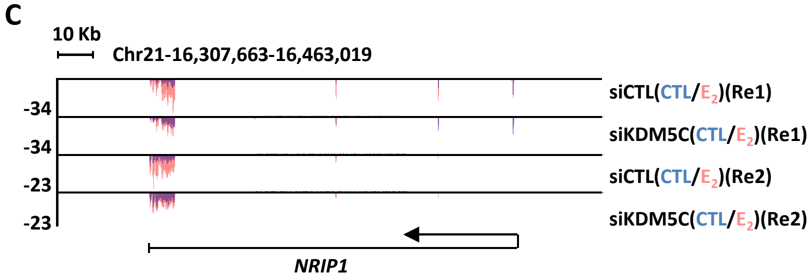

D

siCTL	+	+	–	–	
siKDM5C	–	–	+	+	
E_2	–	+	–	+	
TFF1	1.00, ± 0.00	2.75, ± 0.14	0.41, ± 0.02	0.83, ± 0.07	***
GREB1	1.00, ± 0.00	12.37, ± 0.73	0.54, ± 0.00	3.28, ± 0.22	***
PGR	1.00, ± 0.00	28.73, ± 1.53	0.58, ± 0.14	5.28, ± 0.41	***
NRIP1	1.00, ± 0.00	3.20, ± 0.34	1.28, ± 0.13	2.14, ± 0.17	**
SIAH2	1.00, ± 0.00	8.14, ± 0.26	1.39, ± 0.39	4.07, ± 0.83	***
P2RY2	1.00, ± 0.00	2.30, ± 0.06	1.26, ± 0.07	1.21, ± 0.12	***
RNF223	1.00, ± 0.00	4.67, ± 0.21	1.22, ± 0.20	2.75, ± 0.34	**
MYC	1.00, ± 0.00	7.13, ± 0.64	0.69, ± 0.25	2.43, ± 0.09	***
ADORA1	1.00, ± 0.00	9.85, ± 1.67	0.85, ± 0.10	1.89, ± 0.25	**
KDM5C	1.00, ± 0.00	0.93, ± 0.06	0.34, ± 0.00	0.29, ± 0.01	***

Mean, ± SD

MCF7

E

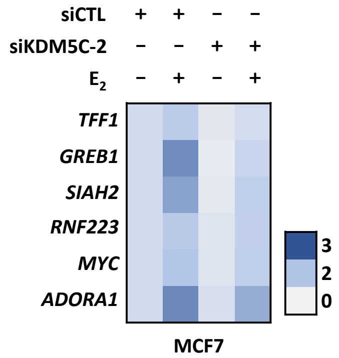

F

siCTL	+	+	–	–	
siKDM5C-2	–	–	+	+	
E_2	–	+	–	+	
TFF1	1.00, ± 0.00	1.68, ± 0.07	0.53, ± 0.01	0.96, ± 0.01	***
GREB1	1.00, ± 0.00	2.46, ± 0.10	0.35, ± 0.01	1.29, ± 0.03	***
SIAH2	1.00, ± 0.00	2.25, ± 0.13	0.51, ± 0.06	1.53, ± 0.08	**
RNF223	1.00, ± 0.00	1.75, ± 0.04	0.61, ± 0.05	1.50, ± 0.03	***
MYC	1.00, ± 0.00	1.88, ± 0.10	0.64, ± 0.02	1.57, ± 0.02	***
ADORA1	1.00, ± 0.00	2.50, ± 0.02	0.83, ± 0.00	2.15, ± 0.07	**

Mean, ± SD

MCF7

G

shCTL	+	+	–	–	
shKDM5C	–	–	+	+	
E_2	–	+	–	+	
TFF1	1.00, ± 0.00	2.95, ± 0.48	0.10, ± 0.01	0.19, ± 0.01	***
GREB1	1.00, ± 0.00	6.29, ± 0.49	0.16, ± 0.01	0.55, ± 0.13	***
PGR	1.00, ± 0.00	23.0, ± 1.91	0.39, ± 0.08	1.25, ± 0.38	***
NRIP1	1.00, ± 0.00	3.12, ± 0.19	1.39, ± 0.14	2.06, ± 0.10	***
SIAH2	1.00, ± 0.00	4.45, ± 0.02	0.35, ± 0.03	0.61, ± 0.02	***
P2RY2	1.00, ± 0.00	1.98, ± 0.24	0.41, ± 0.13	0.65, ± 0.01	***
RNF223	1.00, ± 0.00	2.35, ± 0.01	1.06, ± 0.04	1.49, ± 0.06	***
MYC	1.00, ± 0.00	3.73, ± 0.23	0.23, ± 0.01	0.31, ± 0.02	***
ADORA1	1.00, ± 0.00	2.50, ± 0.49	0.60, ± 0.07	0.82, ± 0.06	**
KDM5C	1.00, ± 0.00	1.18, ± 0.06	0.07, ± 0.01	0.08, ± 0.01	***

Mean, ± SD

MCF7

H

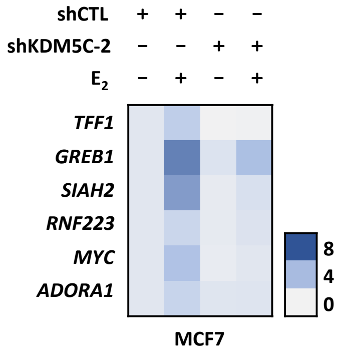

I

shCTL	+	+	–	–	
shKDM5C-2	–	–	+	+	
E_2	–	+	–	+	
TFF1	1.00, ± 0.00	2.95, ± 0.48	0.09, ± 0.00	0.20, ± 0.01	***
GREB1	1.00, ± 0.00	6.29, ± 0.46	1.27, ± 0.03	3.84, ± 0.20	**
SIAH2	1.00, ± 0.00	5.25, ± 0.12	0.68, ± 0.05	1.51, ± 0.04	***
RNF223	1.00, ± 0.00	2.35, ± 0.01	0.69, ± 0.18	1.32, ± 0.13	***
MYC	1.00, ± 0.00	3.73, ± 0.23	0.61, ± 0.10	1.01, ± 0.01	***
ADORA1	1.00, ± 0.00	2.50, ± 0.49	1.11, ± 0.15	1.18, ± 0.08	**

Mean, ± SD

MCF7

Figure S2

J

Gene	WT +, KDM5C (sgRNA) –, E_2 –	WT +, KDM5C (sgRNA) –, E_2 +	WT –, KDM5C (sgRNA) +, E_2 –	WT –, KDM5C (sgRNA) +, E_2 +	
TFF1	1.00, ± 0.00	3.58, ± 0.20	1.24, ± 0.09	1.79, ± 0.14	***
GREB1	1.00, ± 0.00	4.58, ± 0.29	1.07, ± 0.09	2.45, ± 0.22	***
PGR	1.00, ± 0.00	16.15, ± 1.88	1.85, ± 0.10	7.53, ± 1.06	**
NRIP1	1.00, ± 0.00	2.92, ± 0.52	1.11, ± 0.16	1.40, ± 0.07	**
SIAH2	1.00, ± 0.00	3.56, ± 0.25	1.36, ± 0.20	2.61, ± 0.15	**
P2RY2	1.00, ± 0.00	1.38, ± 0.13	1.59, ± 0.03	1.74, ± 0.22	n.s
RNF223	1.00, ± 0.00	1.95, ± 0.03	0.71, ± 0.05	1.56, ± 0.11	**
MYC	1.00, ± 0.00	2.05, ± 0.04	0.43, ± 0.01	0.72, ± 0.06	***
ADORA1	1.00, ± 0.00	5.63, ± 0.72	0.74, ± 0.26	1.36, ± 0.39	***

Mean, ± SD

MCF7

K

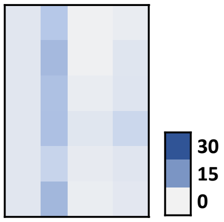

MCF7

L

Gene	WT +, KDM5C (sgRNA-2) –, E_2 –	WT +, KDM5C (sgRNA-2) –, E_2 +	WT –, KDM5C (sgRNA-2) +, E_2 –	WT –, KDM5C (sgRNA-2) +, E_2 +	
TFF1	1.00, ± 0.00	3.39, ± 0.01	0.19, ± 0.00	0.55, ± 0.02	***
GREB1	1.00, ± 0.00	6.54, ± 0.30	0.19, ± 0.01	1.08, ± 0.02	***
SIAH2	1.00, ± 0.00	4.88, ± 0.05	0.55, ± 0.00	1.14, ± 0.04	***
RNF223	1.00, ± 0.00	4.99, ± 0.73	1.03, ± 0.14	2.21, ± 0.26	**
MYC	1.00, ± 0.00	2.46, ± 0.04	0.65, ± 0.00	1.06, ± 0.01	***
ADORA1	1.00, ± 0.00	7.16, ± 0.17	0.46, ± 0.02	0.90, ± 0.21	***

Mean, ± SD

MCF7

M

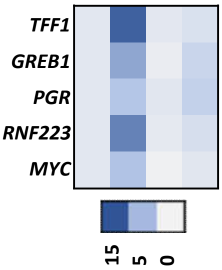

T47D

N

Gene	siCTL +, siKDM5C –, E_2 –	siCTL +, siKDM5C –, E_2 +	siCTL –, siKDM5C +, E_2 –	siCTL –, siKDM5C +, E_2 +	
TFF1	1.00, ± 0.00	13.77, ± 0.59	0.94, ± 0.02	1.50, ± 0.18	***
GREB1	1.00, ± 0.00	6.92, ± 0.12	0.53, ± 0.01	2.43, ± 0.20	***
PGR	1.00, ± 0.00	3.68, ± 0.05	1.06, ± 0.04	2.81, ± 0.19	**
RNF223	1.00, ± 0.00	10.52, ± 0.43	0.82, ± 0.04	1.67, ± 0.07	***
MYC	1.00, ± 0.00	4.00, ± 0.10	0.20, ± 0.02	1.07, ± 0.01	***

Mean, ± SD

T47D

O

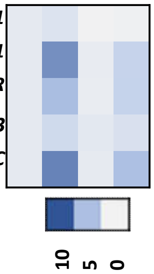

BT474

P

Gene	siCTL +, siKDM5C –, E_2 –	siCTL +, siKDM5C –, E_2 +	siCTL –, siKDM5C +, E_2 –	siCTL –, siKDM5C +, E_2 +	
TFF1	1.00, ± 0.00	1.68, ± 0.13	0.14, ± 0.01	0.31, ± 0.02	***
GREB1	1.00, ± 0.00	7.24, ± 0.21	0.81, ± 0.02	3.37, ± 0.08	***
PGR	1.00, ± 0.00	5.06, ± 0.09	0.72, ± 0.06	3.38, ± 0.08	***
RNF223	1.00, ± 0.00	2.62, ± 0.10	1.15, ± 0.03	1.91, ± 0.00	***
MYC	1.00, ± 0.00	7.80, ± 0.84	0.91, ± 0.06	4.97, ± 0.10	**

Mean, ± SD

BT474

Figure S2 Cont'd

A

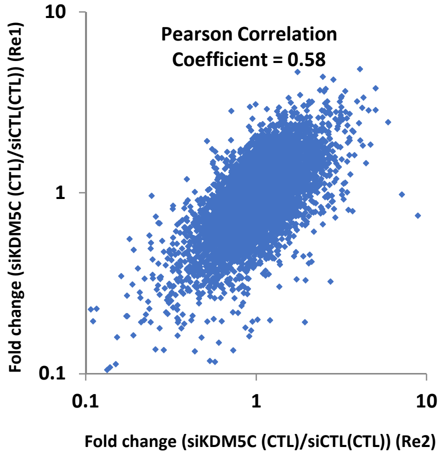

B

	siCTL + / siKDM5C –	siCTL – / siKDM5C +	
IFNβ	1.00, ± 0.00	3.46, ± 0.60	**
IL28A	1.00, ± 0.00	4.39, ± 0.20	****
ISG15	1.00, ± 0.00	2.25, ± 0.17	****
OASL	1.00, ± 0.00	3.28, ± 0.17	****
ISG56	1.00, ± 0.00	2.92, ± 0.07	****
IL6	1.00, ± 0.00	17.09, ± 1.11	****
CCL5	1.00, ± 0.00	3.08, ± 0.05	****
OAS1	1.00, ± 0.00	2.09, ± 0.17	****
IFI27	1.00, ± 0.00	2.32, ± 0.04	****
STAT1	1.00, ± 0.00	1.42, ± 0.18	*

Mean, ± SD

MCF7

C

	shCTL + / shKDM5C –	shCTL – / shKDM5C +	
IFNβ	1.00, ± 0.00	12.69, ± 2.32	***
IL28A	1.00, ± 0.00	3.61, ± 0.42	****
ISG15	1.00, ± 0.00	3.97, ± 0.19	****
OASL	1.00, ± 0.00	7.82, ± 0.36	****
ISG56	1.00, ± 0.00	4.33, ± 0.27	****
IL6	1.00, ± 0.00	117.54, ± 2.86	****
CCL5	1.00, ± 0.00	18.52, ± 0.97	****
OAS1	1.00, ± 0.00	2.66, ± 0.13	****
IFI27	1.00, ± 0.00	2.34, ± 0.35	**
STAT1	1.00, ± 0.00	1.94, ± 0.06	****

Mean, ± SD

MCF7

D

	WT + / KDM5C (sgRNA) –	WT – / KDM5C (sgRNA) +	
IFNβ	1.00, ± 0.00	2.53, ± 0.29	****
IL28A	1.00, ± 0.00	2.52, ± 0.30	****
ISG15	1.00, ± 0.00	2.90, ± 0.21	****
OASL	1.00, ± 0.00	1.57, ± 0.03	****
ISG56	1.00, ± 0.00	2.94, ± 0.14	****
IL6	1.00, ± 0.00	31.02, ± 1.72	****
CCL5	1.00, ± 0.00	1.38, ± 0.08	**
OAS1	1.00, ± 0.00	8.26, ± 0.02	****
IFI27	1.00, ± 0.00	1.43, ± 0.02	****
STAT1	1.00, ± 0.00	1.65, ± 0.06	****

Mean, ± SD

MCF7

E

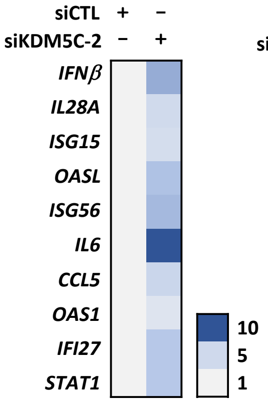

MCF7

F

	siCTL + / siKDM5C-2 –	siCTL – / siKDM5C-2 +	
IFNβ	1.00, ± 0.00	3.82, ± 0.06	***
IL28A	1.00, ± 0.00	1.82, ± 0.10	***
ISG15	1.00, ± 0.00	1.73, ± 0.24	**
OASL	1.00, ± 0.00	2.71, ± 0.12	***
ISG56	1.00, ± 0.00	3.19, ± 0.07	***
IL6	1.00, ± 0.00	8.32, ± 0.45	**
CCL5	1.00, ± 0.00	1.98 ± 0.02	***
OAS1	1.00, ± 0.00	1.50, ± 0.21	*
IFI27	1.00, ± 0.00	2.42, ± 0.17	***
STAT1	1.00, ± 0.00	2.42, ± 0.07	***

Mean, ± SD

MCF7

G

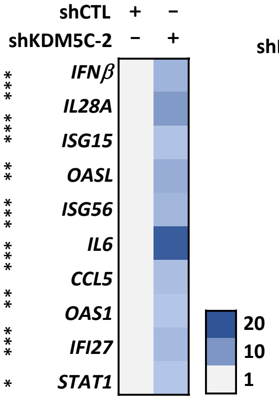

MCF7

H

	shCTL + / shKDM5C-2 –	shCTL – / shKDM5C-2 +	
IFNβ	1.00, ± 0.00	5.46, ± 0.22	***
IL28A	1.00, ± 0.00	9.36, ± 1.10	***
ISG15	1.00, ± 0.00	3.11, ± 0.26	***
OASL	1.00, ± 0.00	6.49, ± 1.19	***
ISG56	1.00, ± 0.00	5.09, ± 0.14	***
IL6	1.00, ± 0.00	18.86, ± 2.26	****
CCL5	1.00, ± 0.00	3.90 ± 0.29	***
OAS1	1.00, ± 0.00	2.78, ± 0.14	***
IFI27	1.00, ± 0.00	4.57, ± 0.16	***
STAT1	1.00, ± 0.00	2.64, ± 0.09	***

Mean, ± SD

MCF7

I

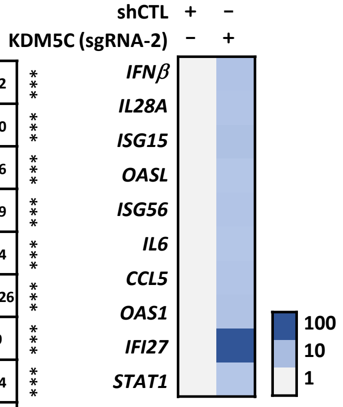

MCF7

J

	WT + / KDM5C (sgRNA-2) –	WT – / KDM5C (sgRNA-2) +	
IFNβ	1.00, ± 0.00	4.78, ± 0.03	***
IL28A	1.00, ± 0.00	3.25, ± 0.54	**
ISG15	1.00, ± 0.00	6.32, ± 0.12	***
OASL	1.00, ± 0.00	1.91, ± 0.16	***
ISG56	1.00, ± 0.00	3.43, ± 0.11	***
IL6	1.00, ± 0.00	1.56, ± 0.28	*
CCL5	1.00, ± 0.00	3.15, ± 0.39	***
OAS1	1.00, ± 0.00	4.95, ± 0.10	***
IFI27	1.00, ± 0.00	99.75, ± 8.66	***
STAT1	1.00, ± 0.00	1.26, ± 0.12	*

Mean, ± SD

MCF7

K

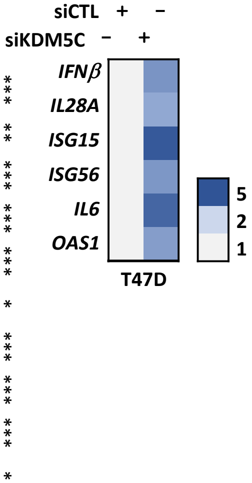

T47D

L

	siCTL + / siKDM5C –	siCTL – / siKDM5C +	
IFNβ	1.00, ± 0.00	3.53, ± 0.13	***
IL28A	1.00, ± 0.00	3.10, ± 0.07	***
ISG15	1.00, ± 0.00	4.83, ± 0.19	***
ISG56	1.00, ± 0.00	3.47, ± 0.10	***
IL6	1.00, ± 0.00	4.55, ± 0.76	**
OAS1	1.00, ± 0.00	3.33, ± 0.09	***

Mean, ± SD

T47D

M

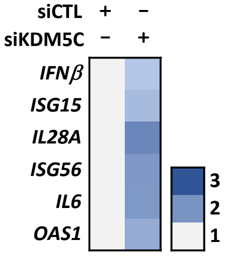

BT474

N

	siCTL + / siKDM5C –	siCTL – / siKDM5C +	
IFNβ	1.00, ± 0.00	1.17, ± 0.10	*
ISG15	1.00, ± 0.00	1.36, ± 0.21	*
IL28A	1.00, ± 0.00	2.20, ± 0.30	**
ISG56	1.00, ± 0.00	1.93, ± 0.39	*
IL6	1.00, ± 0.00	1.91, ± 0.22	**
OAS1	1.00, ± 0.00	1.62, ± 0.02	***

Mean, ± SD

BT474

Figure S3

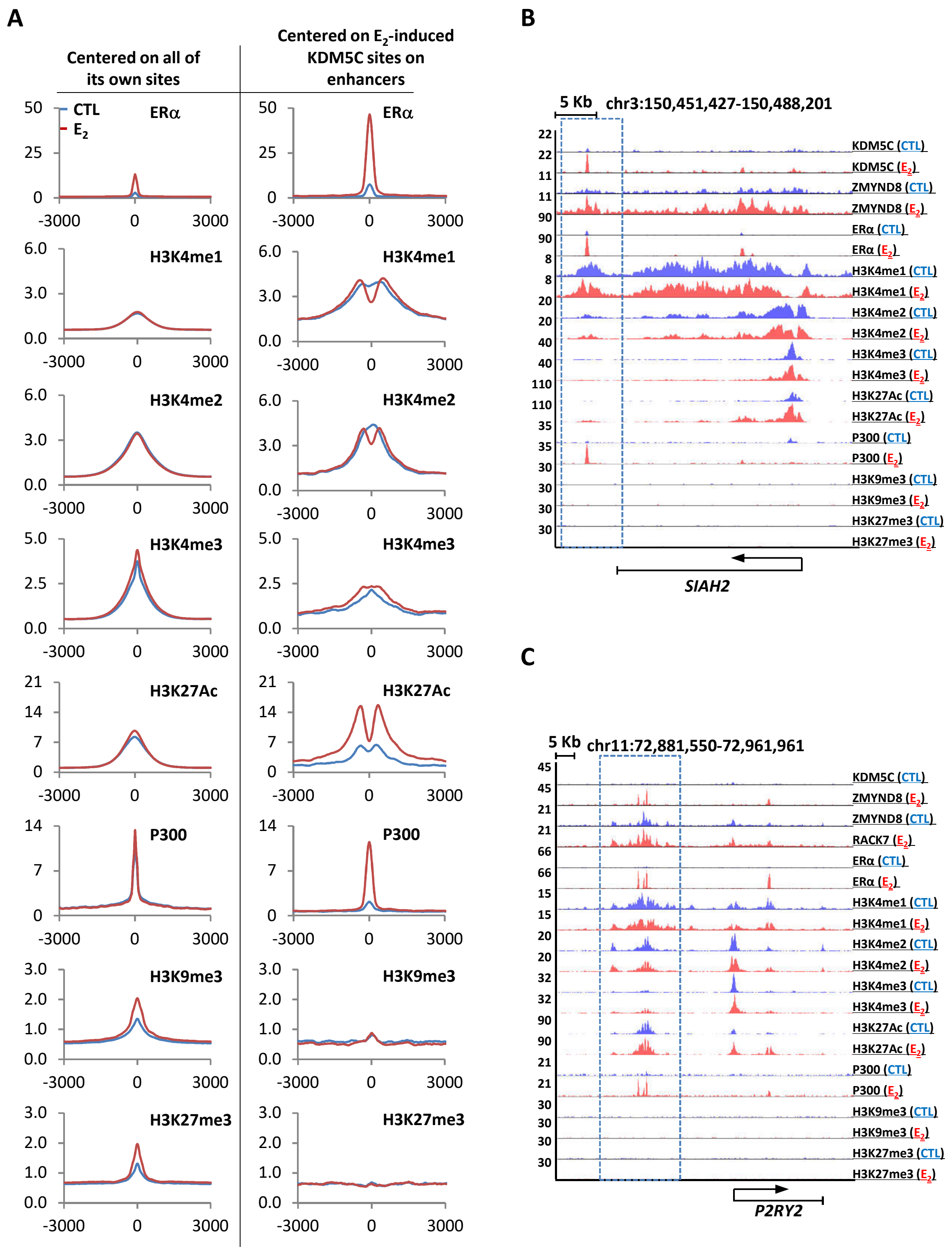
A
Centered on all of its own sites
Centered on E2-induced KDM5C sites on enhancers
CTL
E2
ERα
H3K4me1
H3K4me2
H3K4me3
H3K27Ac
P300
H3K9me3
H3K27me3
B
5 Kb
chr3:150,451,427-150,488,201
KDM5C (CTL)
KDM5C (E2)
ZMYND8 (CTL)
ZMYND8 (E2)
ERα (CTL)
ERα (E2)
H3K4me1 (CTL)
H3K4me1 (E2)
H3K4me2 (CTL)
H3K4me2 (E2)
H3K4me3 (CTL)
H3K4me3 (E2)
H3K27Ac (CTL)
H3K27Ac (E2)
P300 (CTL)
P300 (E2)
H3K9me3 (CTL)
H3K9me3 (E2)
H3K27me3 (CTL)
H3K27me3 (E2)
SIAH2
C
5 Kb
chr11:72,881,550-72,961,961
KDM5C (CTL)
ZMYND8 (E2)
ZMYND8 (CTL)
RACK7 (E2)
ERα (CTL)
ERα (E2)
H3K4me1 (CTL)
H3K4me1 (E2)
H3K4me2 (CTL)
H3K4me2 (E2)
H3K4me3 (CTL)
H3K4me3 (E2)
H3K27Ac (CTL)
H3K27Ac (E2)
P300 (CTL)
P300 (E2)
H3K9me3 (CTL)
H3K9me3 (E2)
H3K27me3 (CTL)
H3K27me3 (E2)
P2RY2

Figure S4

A

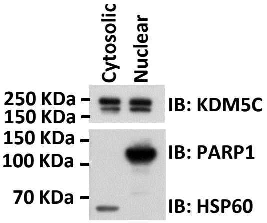

B

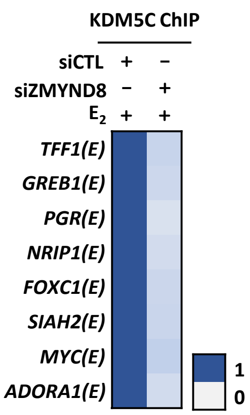

C

KDM5C ChIP

	siCTL + / siZMYND8 – / E2 +	siCTL – / siZMYND8 + / E2 +	
TFF1(E)	1.00, ± 0.00	0.62, ± 0.04	***
GREB1(E)	1.00, ± 0.00	0.56, ± 0.01	***
PGR(E)	1.00, ± 0.00	0.36, ± 0.05	***
NRIP1(E)	1.00, ± 0.00	0.47, ± 0.07	***
FOXC1(E)	1.00, ± 0.00	0.57, ± 0.07	***
SIAH2(E)	1.00, ± 0.00	0.59, ± 0.06	***
MYC(E)	1.00, ± 0.00	0.71, ± 0.02	***
ADORA1(E)	1.00, ± 0.00	0.48, ± 0.01	**

Mean, ± SD

D

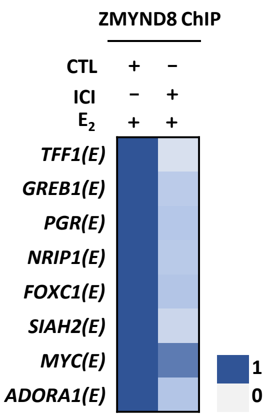

E

ZMYND8 ChIP

	CTL + / ICI – / E2 +	CTL – / ICI + / E2 +	
TFF1(E)	1.00, ± 0.00	0.24, ± 0.03	***
GREB1(E)	1.00, ± 0.00	0.52, ± 0.02	***
PGR(E)	1.00, ± 0.00	0.58, ± 0.04	***
NRIP1(E)	1.00, ± 0.00	0.54, ± 0.09	***
FOXC1(E)	1.00, ± 0.00	0.59, ± 0.00	***
SIAH2(E)	1.00, ± 0.00	0.37, ± 0.01	***
MYC(E)	1.00, ± 0.00	0.86, ± 0.03	**
ADORA1(E)	1.00, ± 0.00	0.59, ± 0.03	***

Mean, ± SD

F

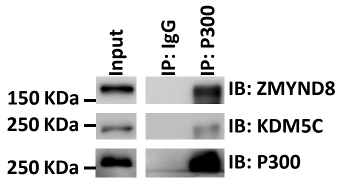

G

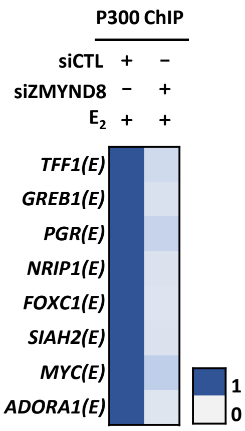

H

P300 ChIP

	siCTL + / siZMYND8 – / E2 +	siCTL – / siZMYND8 + / E2 +	
TFF1(E)	1.00, ± 0.00	0.49, ± 0.15	**
GREB1(E)	1.00, ± 0.00	0.34, ± 0.02	***
PGR(E)	1.00, ± 0.00	0.62, ± 0.08	**
NRIP1(E)	1.00, ± 0.00	0.32, ± 0.05	***
FOXC1(E)	1.00, ± 0.00	0.29, ± 0.03	***
SIAH2(E)	1.00, ± 0.00	0.33, ± 0.02	***
MYC(E)	1.00, ± 0.00	0.72, ± 0.13	***
ADORA1(E)	1.00, ± 0.00	0.27, ± 0.07	***

Mean, ± SD

I

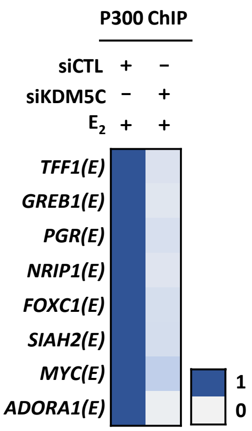

J

P300 ChIP

	siCTL + / siKDM5C – / E2 +	siCTL – / siKDM5C + / E2 +	
TFF1(E)	1.00, ± 0.00	0.33, ± 0.03	***
GREB1(E)	1.00, ± 0.00	0.25, ± 0.01	***
PGR(E)	1.00, ± 0.00	0.37, ± 0.06	***
NRIP1(E)	1.00, ± 0.00	0.27, ± 0.05	***
FOXC1(E)	1.00, ± 0.00	0.40, ± 0.01	***
SIAH2(E)	1.00, ± 0.00	0.41, ± 0.02	***
MYC(E)	1.00, ± 0.00	0.68, ± 0.01	***
ADORA1(E)	1.00, ± 0.00	0.09, ± 0.02	***

Mean, ± SD

Figure S5

A

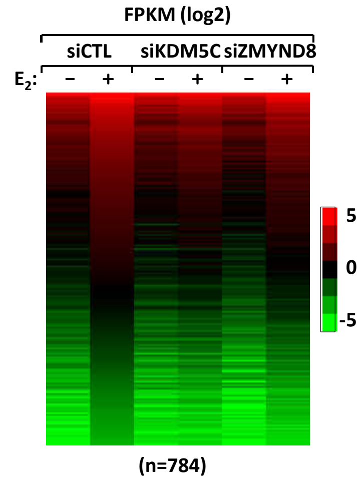

B

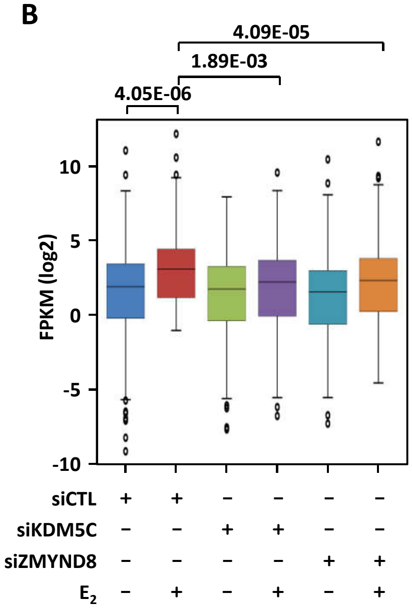

C

MCF7					
siCTL	+	+	–	–	
siZMYND8	–	–	+	+	
E_2	–	+	–	+	
TFF1	1.00, ± 0.00	2.10, ± 0.04	0.65, ± 0.02	1.05, ± 0.11	***
PGR	1.00, ± 0.00	30.55, ± 2.53	1.78, ± 0.28	27.43, ± 5.08	n.s
NRIP1	1.00, ± 0.00	4.75, ± 0.08	1.40, ± 0.06	3.14, ± 0.08	***
P2RY2	1.00, ± 0.00	3.25, ± 0.01	0.81, ± 0.01	1.46, ± 0.15	***
MYC	1.00, ± 0.00	7.02, ± 0.87	0.73, ± 0.12	1.10, ± 0.28	***
ADORA1	1.00, ± 0.00	2.82, ± 0.26	0.41, ± 0.02	0.56, ± 0.21	***

Mean, ± SD

D

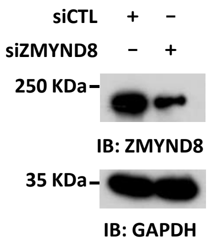

E

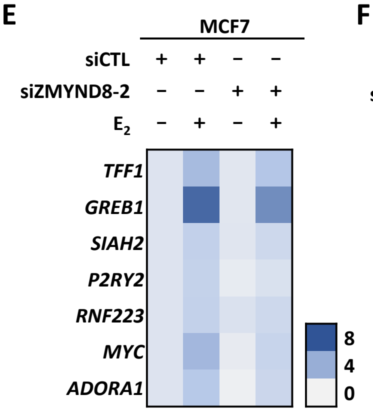

F

MCF7					
siCTL	+	+	–	–	
siZMYND8-2	–	–	+	+	
E_2	–	+	–	+	
TFF1	1.00, ± 0.00	3.44, ± 0.05	0.83, ± 0.01	2.91, ± 0.10	***
GREB1	1.00, ± 0.00	7.13, ± 0.11	0.82, ± 0.01	5.50, ± 0.06	***
SIAH2	1.00, ± 0.00	2.29, ± 0.07	0.88, ± 0.03	1.75, ± 0.04	**
P2RY2	1.00, ± 0.00	2.17, ± 0.04	0.52, ± 0.01	1.18, ± 0.00	***
RNF223	1.00, ± 0.00	2.23, ± 0.06	1.13, ± 0.06	1.77, ± 0.07	***
MYC	1.00, ± 0.00	3.59, ± 0.15	0.55, ± 0.01	2.04, ± 0.18	***
ADORA1	1.00, ± 0.00	2.78, ± 0.27	0.25, ± 0.09	1.87, ± 0.25	**

Mean, ± SD

G

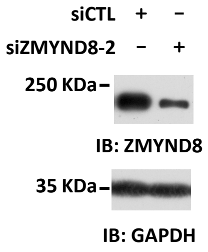

H

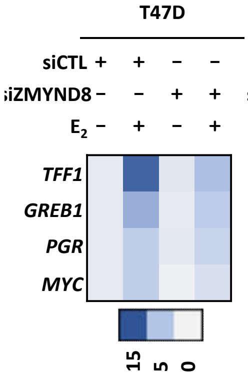

I

T47D					
siCTL	+	+	–	–	
siZMYND8	–	–	+	+	
E_2	–	+	–	+	
TFF1	1.00, ± 0.00	13.66, ± 0.57	1.33, ± 0.07	5.44, ± 0.26	***
GREB1	1.00, ± 0.00	7.04, ± 0.20	0.93, ± 0.03	4.21, ± 0.11	***
PGR	1.00, ± 0.00	4.00, ± 0.09	1.11, ± 0.01	3.32, ± 0.05	***
MYC	1.00, ± 0.00	4.05, ± 0.18	0.40, ± 0.01	2.10, ± 0.04	***

J

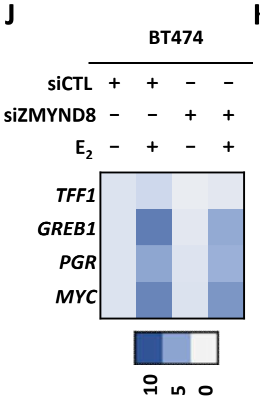

K

BT474					
siCTL	+	+	–	–	
siZMYND8	–	–	+	+	
E_2	–	+	–	+	
TFF1	1.00, ± 0.00	1.66, ± 0.02	0.41, ± 0.02	0.71, ± 0.02	***
GREB1	1.00, ± 0.00	7.41, ± 0.41	0.72, ± 0.05	4.61, ± 0.35	***
PGR	1.00, ± 0.00	4.88, ± 0.15	0.96, ± 0.05	4.20, ± 0.12	**
MYC	1.00, ± 0.00	6.95, ± 0.43	1.05, ± 0.10	5.88, ± 0.08	*

Mean, ± SD

Figure S6

L

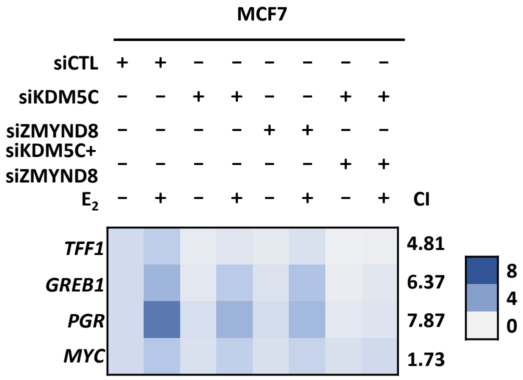

M

MCF7

siCTL	+	+	−	−	−	−	−	−	
siKDM5C	−	−	+	+	−	−	+	+	
siZMYND8	−	−	−	−	+	+	−	−	
siKDM5C+ siZMYND8	−	−	−	−	−	−	+	+	
E_2	−	+	−	+	−	+	−	+	
TFF1	1.00, ± 0.00	1.59, ± 0.07	0.34, ± 0.01	0.60, ± 0.00	0.42, ± 0.00	0.78, ± 0.02	0.16, ± 0.00	0.24, ± 0.00	*** *** ***
GREB1	1.00, ± 0.00	2.81, ± 0.12	0.49, ± 0.01	1.72, ± 0.07	0.72, ± 0.02	2.11, ± 0.07	0.27, ± 0.00	0.52, ± 0.01	*** *** ***
PGR	1.00, ± 0.00	6.02, ± 0.71	0.84, ± 0.16	2.96, ± 0.60	0.92, ± 0.08	2.63, ± 0.28	0.50, ± 0.05	0.63, ± 0.00	** ** ***
MYC	1.00, ± 0.00	1.83, ± 0.06	0.77, ± 0.01	1.53, ± 0.01	0.81, ± 0.03	1.36, ± 0.02	0.79, ± 0.02	1.06, ± 0.04	** *** ***

Mean, ± SD

Figure S6 Cont’d

A

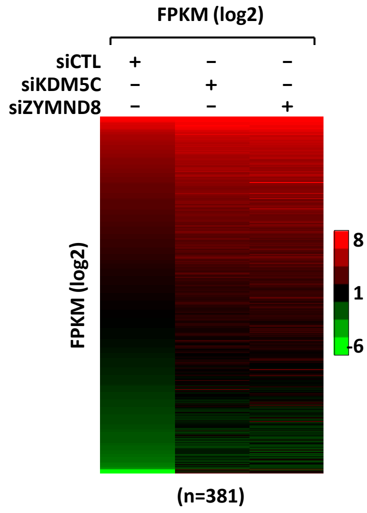

B

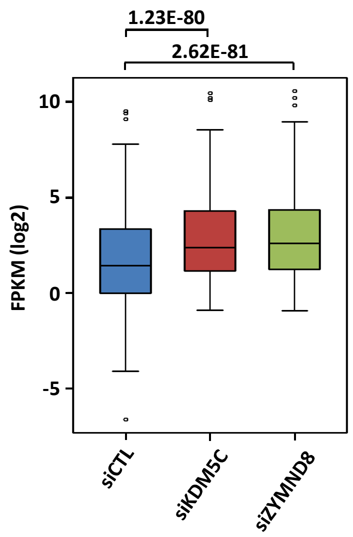

C

MCF7

	siCTL + / siZMYND8 −	siCTL − / siZMYND8 +	
IFNβ	1.00, ± 0.00	2.17, ± 0.52	*
IL28A	1.00, ± 0.00	1.57, ± 0.06	***
ISG15	1.00, ± 0.00	5.21, ± 0.18	***
OASL	1.00, ± 0.00	2.24, ± 0.04	***
ISG56	1.00, ± 0.00	7.16, ± 0.56	***
IL6	1.00, ± 0.00	2.22, ± 0.12	***
CCL5	1.00, ± 0.00	2.24, ± 0.12	***
OAS1	1.00, ± 0.00	5.79, ± 0.49	***
IFI27	1.00, ± 0.00	10.14, ± 1.19	***
STAT1	1.00, ± 0.00	14.07, ± 0.80	***

Mean, ± SD

D

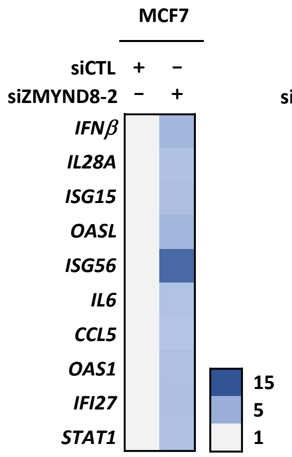

E

MCF7

	siCTL + / siZMYND8-2 − +	siCTL − / siZMYND8-2 + −	
IFNβ	1.00, ± 0.00	3.98, ± 0.10	***
IL28A	1.00, ± 0.00	2.91, ± 0.06	***
ISG15	1.00, ± 0.00	3.17, ± 0.46	**
OASL	1.00, ± 0.00	4.12, ± 0.02	***
ISG56	1.00, ± 0.00	12.77, ± 0.25	***
IL6	1.00, ± 0.00	2.81, ± 0.25	**
CCL5	1.00, ± 0.00	2.36 ± 0.06	***
OAS1	1.00, ± 0.00	3.02, ± 0.09	***
IFI27	1.00, ± 0.00	3.14, ± 0.04	***
STAT1	1.00, ± 0.00	2.93, ± 0.03	***

Mean, ± SD

F

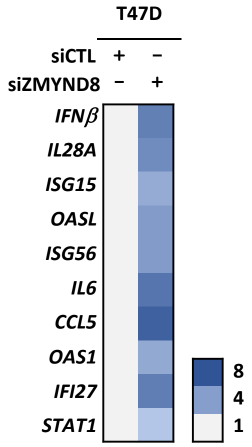

G

T47D

	siCTL + / siZMYND8 −	siCTL − / siZMYND8 +	
IFNβ	1.00, ± 0.00	5.63, ± 0.21	***
IL28A	1.00, ± 0.00	5.01, ± 0.06	***
ISG15	1.00, ± 0.00	3.28, ± 0.11	***
OASL	1.00, ± 0.00	4.15, ± 0.22	***
ISG56	1.00, ± 0.00	5.13, ± 0.09	***
IL6	1.00, ± 0.00	6.21, ± 0.27	***
CCL5	1.00, ± 0.00	7.33 ± 0.17	***
OAS1	1.00, ± 0.00	3.40, ± 0.03	***
IFI27	1.00, ± 0.00	5.74, ± 0.19	***
STAT1	1.00, ± 0.00	1.77, ± 0.07	***

Mean, ± SD

Figure S7

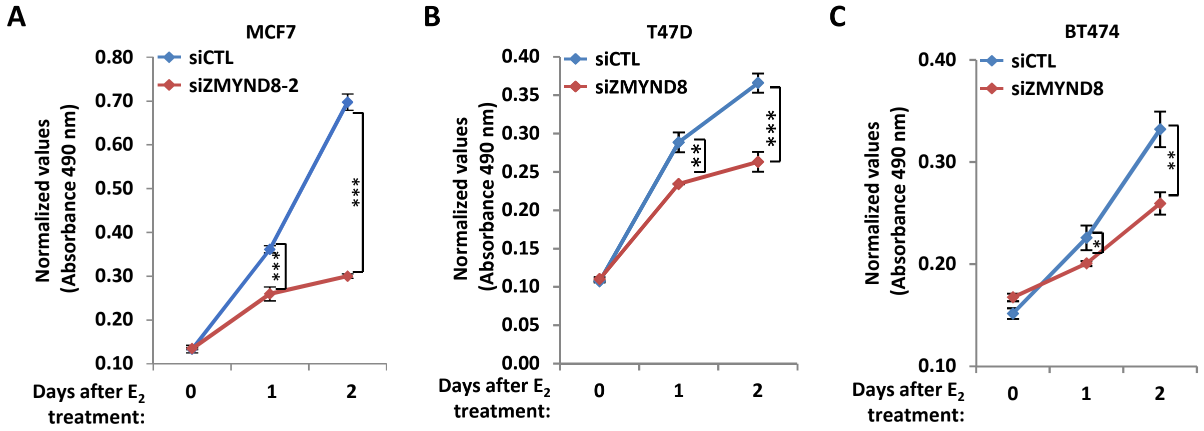
A
MCF7
siCTL
siZMYND8-2
Normalized values (Absorbance 490 nm)
0.80
0.70
0.60
0.50
0.40
0.30
0.20
0.10
***
***
Days after E2 treatment:
0
1
2
B
T47D
siCTL
siZMYND8
0.40
0.35
0.30
0.25
0.20
0.15
0.10
0.05
0.00
**
***
C
BT474
siCTL
siZMYND8
0.40
0.30
0.20
0.10
*
**

Figure S8

A

H3K4me2 occupancy

siCTL	+	+	−	−
siKDM5C	−	−	+	+
E_2	−	+	−	+
TFF1 (E)	1.00, ± 0.00	0.79, ± 0.01	0.99, ± 0.07	0.69, ± 0.05
GREB1 (E)	1.00, ± 0.00	0.55, ± 0.04	0.63, ± 0.05	0.52, ± 0.05
NRIP1 (E)	1.00, ± 0.00	0.65, ± 0.02	0.96, ± 0.07	0.60, ± 0.04

Mean, ± SD

B

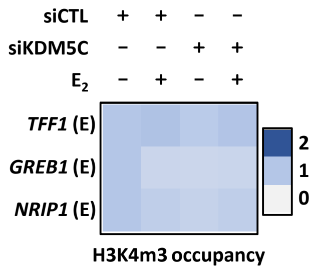

C

H3K4me3 occupancy

siCTL	+	+	−	−
siKDM5C	−	−	+	+
E_2	−	+	−	+
TFF1 (E)	1.00, ± 0.00	1.04, ± 0.12	0.91, ± 0.11	1.02, ± 0.13
GREB1 (E)	1.00, ± 0.00	0.66, ± 0.07	0.64, ± 0.11	0.66, ± 0.11
NRIP1 (E)	1.00, ± 0.00	0.84, ± 0.04	0.74, ± 0.03	0.83, ± 0.22

Mean, ± SD

D

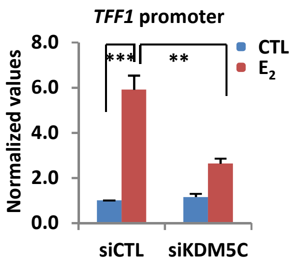

E

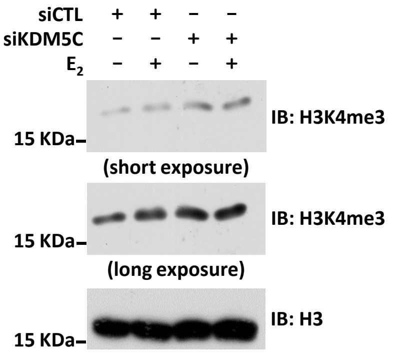

F

CTL	+	+	−	−
E_2	−	+	−	+
C70	−	−	+	+
TFF1	1.00, ± 0.00	2.47, ± 0.08	0.73, ± 0.08	1.85, ± 0.06
GREB1	1.00, ± 0.00	4.24, ± 0.11	0.55, ± 0.02	3.70, ± 0.24
PGR	1.00, ± 0.00	11.95, ± 1.73	1.92, ± 0.32	17.23, ± 2.61
NRIP1	1.00, ± 0.00	2.98, ± 0.24	1.45, ± 0.02	4.43, ± 0.47
SIAH2	1.00, ± 0.00	4.32, ± 0.27	1.08, ± 0.04	3.90, ± 0.31
P2RY2	1.00, ± 0.00	1.62, ± 0.05	0.96, ± 0.04	1.74, ± 0.13
RNF223	1.00, ± 0.00	3.22, ± 0.54	0.80, ± 0.05	1.93, ± 0.12
MYC	1.00, ± 0.00	2.43, ± 0.03	1.07, ± 0.04	1.91, ± 0.17
ADORA1	1.00, ± 0.00	4.17, ± 0.72	2.02, ± 0.24	4.16, ± 0.23

Mean, ± SD

G

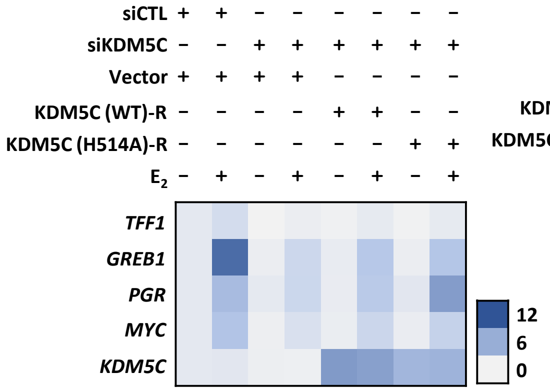

H

siCTL	+	+	−	−	−	−	−	−	
siKDM5C	−	−	+	+	+	+	+	+	
Vector	+	+	+	+	−	−	−	−	
KDM5C (WT)-R	−	−	−	−	+	+	−	−	
KDM5C (H514A)-R	−	−	−	−	−	−	+	+	
E_2	−	+	−	+	−	+	−	+	
TFF1	1.00, ± 0.00	2.20, ± 0.08	0.07, ± 0.00	0.54, ± 0.02	0.22, ± 0.01	0.87, ± 0.00	0.17, ± 0.00	0.85, ± 0.04	*** *** ns
GREB1	1.00, ± 0.00	10.45, ± 0.32	0.52, ± 0.02	2.68, ± 0.12	0.73, ± 0.07	4.18, ± 0.11	0.53, ± 0.03	4.33, ± 0.22	*** *** ns
PGR	1.00, ± 0.00	5.07, ± 0.60	0.96, ± 0.18	2.72, ± 0.17	0.70, ± 0.33	3.94, ± 0.39	1.18, ± 0.41	7.16, ± 1.18	** ** *
MYC	1.00, ± 0.00	4.49, ± 0.11	0.41, ± 0.01	1.81, ± 0.17	0.58, ± 0.02	2.77, ± 0.00	0.60, ± 0.01	3.20, ± 0.15	*** *** **
KDM5C	1.00, ± 0.00	1.19, ± 0.07	0.43, ± 0.02	0.39, ± 0.03	7.33, ± 0.14	6.94, ± 0.26	5.42, ± 0.19	5.65, ± 0.11	

Mean, ± SD

I

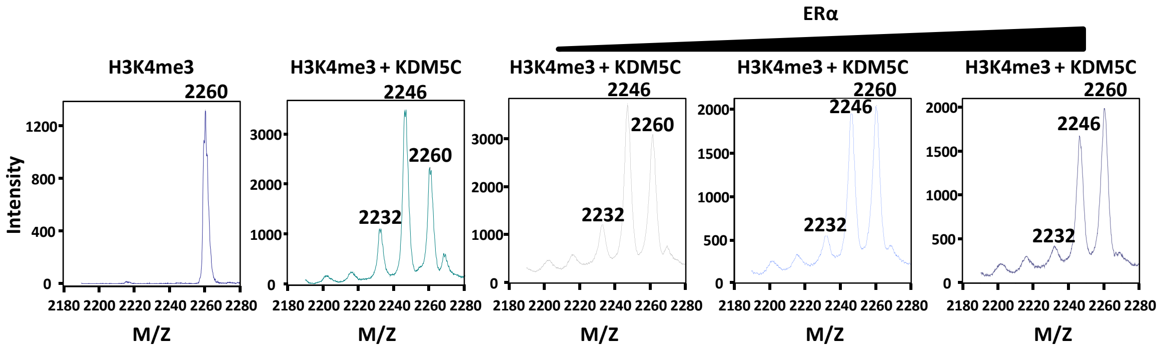

Figure S9

A

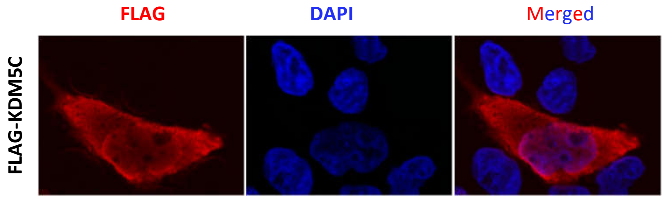

B

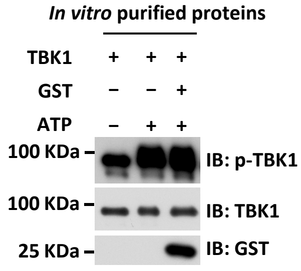

C

siCTL	+	–	–	–			
siKDM5C	–	+	+	+			
Vector	+	+	–	–			
KDM5C (WT)-R	–	–	+	–			
KDM5C (H514A)-R	–	–	–	+			
IFNβ	1.00, ± 0.00	7.40, ± 0.39	1.75, ± 0.17	3.15, ± 0.11	***	***	***
IL28A	1.00, ± 0.00	7.39, ± 0.39	2.23, ± 0.25	3.97, ± 0.22	***	***	***
OASL	1.00, ± 0.00	3.80, ± 0.44	1.51, ± 0.05	2.58, ± 0.33	***	***	**
ISG56	1.00, ± 0.00	4.26, ± 0.33	1.73, ± 0.07	2.19, ± 0.13	***	***	***
IL6	1.00, ± 0.00	9.32, ± 0.61	1.44, ± 0.29	2.68, ± 0.49	***	***	***
CCL5	1.00, ± 0.00	2.46, ± 0.03	1.09, ± 0.04	1.24, ± 0.00	***	***	**
STAT1	1.00, ± 0.00	2.46, ± 0.14	2.01, ± 0.09	2.36, ± 0.05	***	**	**

Mean, ± SD

Figure S10

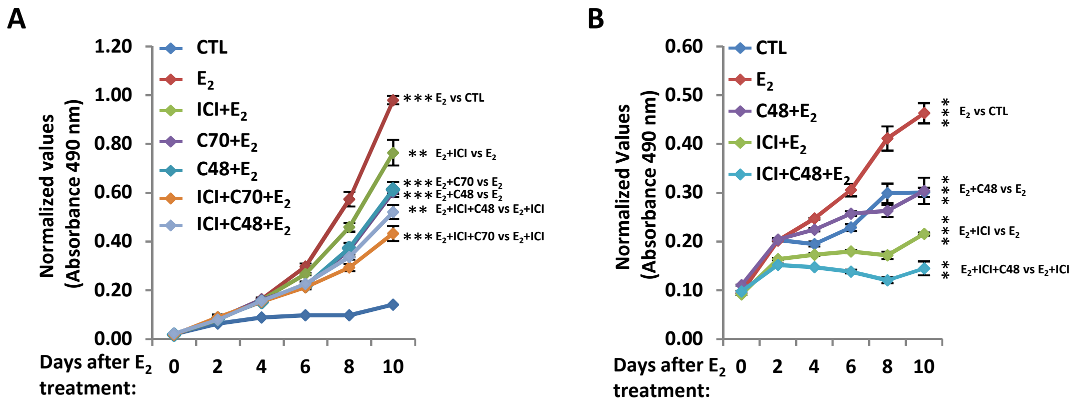
A
B
Normalized values (Absorbance 490 nm)
Normalized Values (Absorbance 490 nm)
Days after E2 treatment:
CTL
E2
ICI+E2
C70+E2
C48+E2
ICI+C70+E2
ICI+C48+E2
*** E2 vs CTL
** E2+ICI vs E2
*** E2+C70 vs E2
*** E2+C48 vs E2
** E2+ICI+C48 vs E2+ICI
*** E2+ICI+C70 vs E2+ICI
E2+C48 vs E2
E2+ICI vs E2
E2+ICI+C48 vs E2+ICI

Figure S11

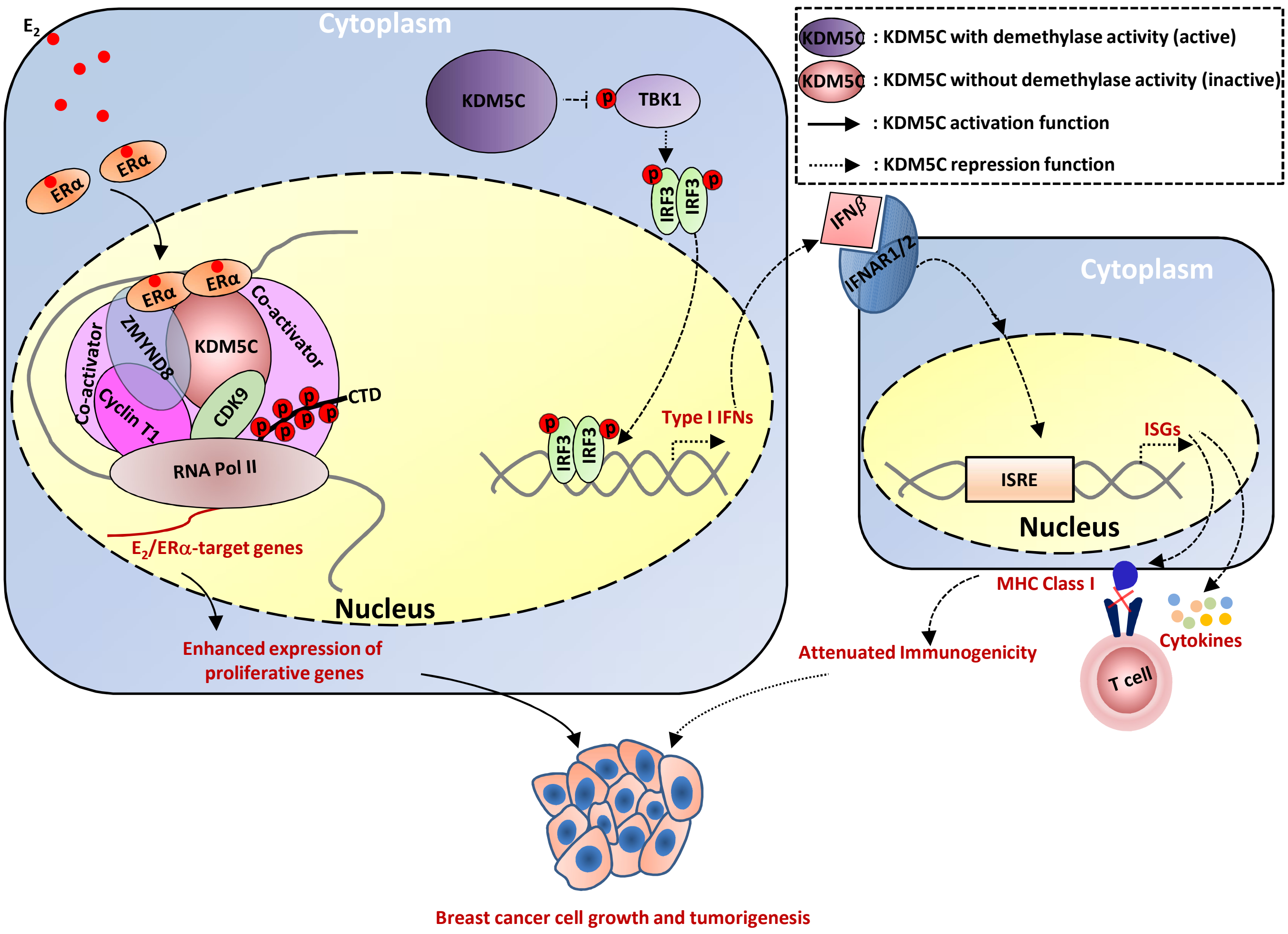

Cytoplasm
E_2
ERα
ERα
KDM5C
p
TBK1
p
IRF3
IRF3
p
Co-activator
ZMYND8
KDM5C
Co-activator
Cyclin T1
CDK9
p
p
p
p
p
p
CTD
RNA Pol II
E_2/ERα-target genes
Nucleus
Enhanced expression of proliferative genes
p
IRF3
IRF3
p
Type I IFNs
KDM5C : KDM5C with demethylase activity (active)
KDM5C : KDM5C without demethylase activity (inactive)
: KDM5C activation function
: KDM5C repression function
IFNβ
IFNAR1/2
Cytoplasm
ISGs
ISRE
Nucleus
MHC Class I
Attenuated Immunogenicity
Cytokines
T cell
Breast cancer cell growth and tumorigenesis

Figure S12
